# Integrated Transcriptomic and Metabolomic Analyses Reveal Acyl-CoA Dehydrogenase-Mediated Primordium Formation and Metabolite Synthesis in *Cordyceps militaris*

**DOI:** 10.3390/jof12090696

**Published:** 2026-09-17

**Authors:** Xin Zhou, Luman Xue, Yuchen Luo, Xinxin Tong, Jinlin Guo

**Affiliations:** Key Laboratory of Standardization of Chinese Medicine, State Key Laboratory of Southwestern Chinese Medicine Resources, School of Pharmacy, Chengdu University of Traditional Chinese Medicine, Chengdu 611137, China; xinzhou@stu.cdutcm.edu.cn (X.Z.); xueluman@stu.cdutcm.edu.cn (L.X.); luosensei@stu.cdutcm.edu.cn (Y.L.)

**Keywords:** *Cordyceps militaris*, acyl-CoA dehydrogenase, transcriptome, untargeted metabolomics, PR development, bioactive nucleosides

## Abstract

*Cordyceps militaris* is an edible entomopathogenic fungus with substantial nutritional and medicinal value. However, the molecular mechanisms underlying the vegetative mycelia-to-primordia (PR) transition and bioactive metabolite synthesis are still poorly defined. Deletion of the acyl-CoA dehydrogenase gene (*ACAD*) caused malformed, stunted PR-like structures together with excessive aerial mycelial growth, severely disrupting PR initiation and fruiting-body elongation in *C. militaris*. Transcriptomic, untargeted metabolomic and multi-omics analyses were performed on wild-type (WT) and *ACAD*-knockout strains at the mycelial and PR stages to explore gene expression reprogramming and bioactive nucleoside accumulation during the developmental transition and under *ACAD* deficiency. In total, 2014, 448, and 838 differentially expressed genes (DEGs) were identified in WT PR vs. WT mycelia, Δ*ACAD* mycelia vs. WT mycelia, and Δ*ACAD* PR vs. WT PR, demonstrating extensive transcriptional reprogramming during the development switch and upon *ACAD* deletion. KEGG enrichment and WGCNA analyses showed that *ACAD* deficiency significantly suppresses multiple reproductive-development pathways, including cell cycle progression, purine biosynthesis, the TCA cycle, MAPK signaling, redox homeostasis, fatty acid and amino acid metabolism. Consistently, *ACAD* deletion markedly increased intracellular ROS level and substantially reduced cellular ATP levels in mycelia. Several crucial genes modulated by *ACAD* were identified, including GTP cyclohydrolase I, histone lysine N-methyltransferase ASHR1, adenine phosphoribosyl transferase 1, glutathione-S-transferase F3 and CoA-ligase CCL8. Multi-omics integration demonstrated tightly coordinated transcriptional and metabolic regulation dependent on *ACAD* function. *ACAD* deficiency significantly disturbed amino acid, lipid, and nucleotide metabolism and reduced the abundance of amino acid derivatives, lipid compounds and purine nucleoside precursors in mycelia. Particularly, adenosine analogs serve as key precursors of the core bioactive nucleoside cordycepin. Key DEGs, including peroxisomal (S)-2-hydroxyacid oxidase, isocitrate lyase 2 and L-ascorbate peroxidase 5, were linked to these metabolic shifts. Our findings demonstrate that *ACAD* serves as an essential regulator that interconnects carbon metabolism, redox homeostasis, lipid, amino acid and purine metabolism to promote PR formation and secondary metabolite synthesis in *C. militaris*.

## 1. Introduction

*Cordyceps militaris* is a well-known ethnomedicinal fungus that has been widely utilized as a traditional crude drug and a functional food in Asia [1]. It contains a broad spectrum of bioactive compounds, including cordycepin (COR), pentostatin (PTN), adenosine, polysaccharides, carotenoids and ergosterol, which exhibit immunomodulatory, anti-inflammatory, antitumor, and antimicrobial activities [1]. Recent advances in synthetic biology and metabolic engineering have enabled the engineering of microbial cell factories such as *Escherichia coli*, *Saccharomyces cerevisiae*, and *Corynebacterium glutamicum* to produce high-value chemicals for industrial application [2]. As an emerging fungal chassis, *C. militaris* exhibits great potential for in-depth fundamental research and synthetic engineering [3]. Fruiting bodies are the principal tissue accumulating bioactive metabolites. Primordium (PR) formation is the initial and critical stage of fruiting-body development. Elucidating the molecular mechanisms underlying PR development and metabolite biosynthesis is critical for the metabolic engineering of *C. militaris.*

Acyl-CoA dehydrogenase (*ACAD*), a member of the acyl-CoA dehydrogenase superfamily, catalyzes the initial step of mitochondrial fatty acid β-oxidation (FAO), a core catabolic pathway that fuels the respiratory chain and maintains cellular energy homeostasis in aerobic organisms [4]. Accumulating evidence underscores its indispensable role in fungal fruiting-body morphogenesis [5]. For instance, lipid accumulation takes place in the specialized hyphae phase preceding perithecial PR; these stored lipids are subsequently oxidized by FAO together with the glyoxylate cycle to generate energy and intermediates that support sexual fruiting-body formation in *Gibberella zeae* [6]. Consistently, the glyoxylate shunt is required for perithecia formation, which is presumably linked to the remobilization of storage lipids. Deletion of the gene encoding pheromone response transcription factor 1 (*SsPRF1*) abolishes mating competence, filamentous growth and pathogenicity in *Sporisorium scitamineum*, highlighting its central regulatory role in sexual reproduction and virulence [7]. Recent research has revealed that the cytochrome P450 gene *CYP8* modulates the transcription of *SsPRF1* through fatty acid metabolic pathways, which ultimately governs sexual development in *S. scitamineum* [8]. Together, *ACAD*-mediated FAO substantially contributes to fungal sexual development and metabolite production.

*ACAD* directly modulates the intracellular acetyl-CoA pool through the FAO pathway [9]. As a core metabolic intermediate, acetyl-CoA governs the catabolism of non-fermentable carbon sources and further regulates secondary metabolite (SM) biosynthesis, fungal sexual development, and stress resistance [9]. It acts as the sole acetyl donor for protein acetylation, thereby fine-tuning protein function at the post-translational level [10,11]. Moreover, the acetyl-CoA metabolic checkpoint has emerged as a key regulatory module through which post-translational modifications (PTMs) control carbon flux partitioning, providing a minimally disruptive strategy for engineering fungal cell factories [9,10,12]; e.g., the acetyl-CoA-dependent checkpoint centered on O-methyltransferase CCM_06472 enables a >4-fold elevation in cordycepin production through a single PTM-mimetic mutation in this regulatory module [13]. Collectively, the *ACAD*-mediated intracellular acetyl-CoA pool facilitates fungal reproduction and SM biosynthesis.

In addition, *ACAD* plays a fundamental role in oxidative stress response; e.g., *ACAD*-disrupted strains exhibit higher sensitivity to oxidative stress than WT strains in *Magnaporthe oryzae* [14]. Enzymes involved in FAO mitigate oxidative stress in tissues by preventing the overaccumulation of free radicals derived from lipid peroxidation [14]. Cumulative evidence demonstrated that ROS homeostasis is required for normal sexual development in fungi; e.g., excessive ROS induces lipid peroxidation and compromises cell wall integrity (CWI), thereby hindering the differentiation of PR and perithecial formation [15]. Loss of carnitine acetyltransferase activity impairs lipid catabolism and acetyl-CoA shuttling, disturbs intracellular ROS homeostasis, and markedly represses perithecium development [16].

*ACAD* participates in maintaining CWI, which is tightly relevant to fungal development via cell wall biogenesis and remodeling of cell-surface properties [17,18]. Moreover, *ACAD* plays a crucial and functionally complementary role in promoting redox homeostasis in *M. oryzae* through enforcing CWI [17]. Together, *ACAD*-mediated FAO supports ROS homeostasis, thereby reinforcing CWI to promote fungal sexual development. In our previous study, integrated quantitative proteomics and lysine succinylation (Ksucc) proteomics showed that the protein abundance of *ACAD* is significantly upregulated during PR formation compared to the sclerotia stage in *Ophiocordyceps sinensis.* This expression change is accompanied by enhanced Ksucc modification of related proteins, indicating that protein Ksucc modification activates *ACAD* to initiate PR formation. Nevertheless, systematic investigations into roles are scarce, and molecular mechanisms of *ACAD*-governed fruiting-body development and SM synthesis remain largely uncharacterized in fungi.

With the advancement of high-throughput sequencing and metabolomics technologies, multi-omics integration has emerged as a powerful strategy for elucidating fungal developmental differentiation and metabolic regulatory mechanisms [19]. Transcriptomics captures dynamic changes in gene expression, whereas metabolomics reflects downstream metabolic responses. Their integration provides a comprehensive view from gene regulation to metabolic phenotype, helping to uncover the molecular basis of biological processes. In the present study, functional verification, transcriptome sequencing, and untargeted metabolomics were integrated to systematically explore gene function, compare differential gene expression patterns and metabolite profiles, and construct transcriptome–metabolome association networks in WT and *ACAD*-deletion (Δ*ACAD*) strains of *C. militaris* at the mycelial and PR stages. Our study aims to reveal the molecular mechanisms by which *ACAD* regulates PR formation and SM synthesis, providing a theoretical basis for the cultivation optimization and genetic engineering improvement of *C. militaris*.

## 2. Materials and Methods

### 2.1. Strains, Plasmids and Media

*C. militaris* strain CICC237586 was provided by the Center for Mushroom Spawn Standards and Control of China (CCMSSC, Beijing, China). The strain was cultured on peptone potato dextrose agar (PPDA) slants containing 2.4% (*w*/*v*) potato dextrose broth and 2% (*w*/*v*) agar (BD Difco, Franklin Lakes, NJ, USA) in the dark for 14 days. Subsequently, approximately 5 mL of conidia suspension (1 × 10^7^ conidia/mL) was harvested and inoculated into a wheat-based medium consisting of 18 g wheat grains, 2 g silkworm pupae powder and 33 mL ddH_2_O. The cultures were incubated in an illumination incubator (MGC-450BP, Yiheng, Shanghai, China) with a 12 h/12 h light/dark (LD) photoperiod for 12 days prior to sequencing and quantitative assays. For PR and fruiting-body development, cultures were cultivated for 15 and 40 days, respectively. The vertical distance between LED light sources and agar plates was set to 50 cm, with a light intensity of 500 lux. The culture temperature was kept constant at 22 °C.

*Escherichia coli* DH5α (TsingKe Biotechnology, Beijing, China) and *Agrobacterium tumefaciens* AGL-1 were cultured in Luria–Bertani (LB) broth (1% NaCl, 0.5% yeast extract, 1% tryptone) or on LB agar for plasmid construction and propagation. Induction medium and co-cultivation medium (IMAS) were applied for *A. tumefaciens*-mediated transformation (ATMT) of *C. militaris.* All microbial strains and plasmids utilized in this study are summarized in Table S1.

### 2.2. Construction of C. militaris ACAD Knockout and Complementation Strains

The gene deletion vector pEX4 and complementation vector pCT741 were kindly provided as a gift by Prof. Richou Han (Guangdong Key Laboratory of Animal Conservation and Resource Utilization, Guangzhou, China). To characterize the function of the *ACAD* gene (Gene ID: CCM_03805), the *C. militaris* Δ*ACAD* knockout mutant and complementary strain were constructed in this study. Fungal genomic DNA was isolated using the standard cetyltrimethylammonium bromide (CTAB) method [20].

To generate the Δ*ACAD* mutant, the recombinant knockout plasmid pEX4-hpt-*ACAD* was constructed, verified by Sanger sequencing and then transformed into *A. tumefaciens* strain AGL-1 [21]. Transformants were screened on PPDA agar plates containing 800 μg/mL hygromycin B at 22 °C. For genetic complementation, the complementation plasmid pCT741-Neo-*ACAD* was constructed and subsequently introduced into the Δ*ACAD* strain through PEG-mediated protoplast transformation [22]. Transformants were screened on PPDA agar plates containing 2000 μg/mL G418 and 800 μg/mL hygromycin B at 22 °C.

Putative transformants of knockout and complemented strains were verified by PCR. Primers used in this work are summarized in Table S1, and agarose gel electrophoresis validation results for plasmid construction are presented in Figure S1. Detailed protocols for fragment amplification, vector linearization, plasmid assembly, and transformant screening are described in Table S1.

### 2.3. Transcriptome Sequencing and Analysis

WT and *ACAD*-knockout strains at the mycelial and PR stages were subjected to transcriptomic sequencing and analysis. Total RNA was extracted from tissue samples using TRIzol reagent (Invitrogen, Carlsbad, CA, USA). The integrity and purity of the extracted RNA were evaluated using a NanoDrop 2000 spectrophotometer and the Agilent 2100 Bioanalyzer (Agilent Technologies, Santa Clara, CA, USA), with the RNA Integrity Number (RIN) required to be greater than 7.0. Transcriptome sequencing and analysis were conducted by OE Biotech Co., Ltd. (Shanghai, China). mRNA was first enriched via poly(A) selection, then fragmented and reverse-transcribed into double-stranded cDNA. cDNA libraries were prepared via cDNA end-polishing, adapter ligation, and fragment size selection; 150 bp paired-end sequencing was carried out on an Illumina NovaSeq sequencing system (Illumina, Inc., San Diego, CA, USA). Sequencing quality control parameters, including sequencing depth, clean read count, Q20, Q30 and GC content, were calculated for each library. The reference-genome index was built with HISAT2 (v2.1.0), and gene-expression levels were quantified and normalized as FPKM values. The raw data of *C. militaris* was submitted to the Genome Sequence Archive (GSA, https://ngdc.cncb.ac.cn/gsub/, accessed on 2 August 2026) as BioProjectPRJNA027168. DEGs were performed from three pairwise comparisons: CMP vs. CMM (WT PR vs. WT mycelia), CMKM vs. CMM (Δ*ACAD* mycelia vs. WT mycelia), CMKP vs. CMP (Δ*ACAD* PR vs. WT PR). Differential expression analysis was conducted using DESeq (v1.38.3), with screening for DEGs with |log_2_FC| > 1 and *FDR* < 0.01. Statistical enrichment of DEGs in the KEGG pathways was analyzed using cluster Profiler (v4.6.0), with a significance threshold of *p* < 0.05. Functional annotation was implemented via sequence homology searches against public databases, namely NR, Swiss-Prot, Gene Ontology (GO), Kyoto Encyclopedia of Genes and Genomes (KEGG) and Pfam. For functional enrichment analysis of DEGs, only terms with a q-value ≤ 0.001 were regarded as significantly enriched in GO and KEGG pathways.

WGCNA v1.69 package in R (v4.2.1) was employed to conduct a weighted correlation analysis based on all genes [23]. An adjacency matrix was calculated with the optimal soft-thresholding power (β) and transformed into a topological overlap matrix (TOM), which was further converted into a distance matrix to build a tree of gene clusters. A dynamic cutting algorithm was applied to cluster all genes into color-coded modules. To identify modules that are strongly related to phenotypic traits, Pearson’s correlation coefficients (R ≥ 0.75, *p* ≤ 0.05) were calculated between each module and each sample, with the parameters set to default values. A heatmap was drawn according to these correlation coefficients, allowing for the selection of phenotype-related modules. A gene co-expression network was constructed, with a focus on edges with a weight > 0.3. The co-expression network of the DEGs in each sample could be constructed by Cytoscape v3.10.1. The minimum required interaction score of confidence was set to be 0.4. Hub genes in the network were identified based on the degree of centrality [24].

### 2.4. Metabolomic Analysis

Metabolomic profiling and data analysis were performed using the standard untargeted metabolomics pipeline of Shanghai Luming Biological Technology Co., Ltd. (Shanghai, China). The preparation and extraction analysis of metabolites followed the method with modifications [25]. Briefly, approximately 50 mg of each sample was extracted with 400 μL of water/acetonitrile/isopropanol (1:1:1, *v*/*v*/*v*). After pre-cooling at −40 °C for 2 min, samples were homogenized at 45 Hz for 60 s, ultrasonicated in an ice-water bath for 30 min, and incubated at −40 °C overnight. After centrifugation at 12,000 rpm for 10 min at 4 °C, 150 μL of the supernatant was transferred to UPLC-MS vials for analysis.

Metabolomic data were acquired using a UPLC system (Vanquish UPLC, Thermo Fisher Scientific, Waltham, MA, USA) coupled with a high-resolution mass spectrometer (Q Exactive HFX, Thermo Fisher Scientific, Waltham, MA, USA). Chromatographic separation was performed on a Waters HSS T3 column (100 mm × 2.1 mm, 1.8 μm), with a binary mobile phase comprising 0.1% formic acid in water (A) and 0.1% formic acid in acetonitrile (B). Electrospray ionization (ESI) was used for ion generation, and MS was recorded in both positive and negative ion modes. Quality control (QC) samples were inserted at regular intervals throughout the analytical sequence to monitor system stability and data reliability.

Raw MS data were preprocessed via Progenesis QI software (V2.3, Nonlinear, Dynamics, Newcastle, UK), covering baseline correction, peak detection, matching, retention time calibration and peak alignment. Metabolite identification was achieved by matching experimental MS/MS spectra against a self-built database, and annotations with matching scores above 0.5 were regarded as high-confidence identifications.

Metabolic features with zero values in over 80% of samples were excluded. Residual data were normalized using the metNor function of the R-based MetNormalizer package, followed by QC filtering to remove metabolites with relative standard deviation (RSD) > 30% in QC samples. PCA was conducted with unit variance scaling, while OPLS-DA was performed with Pareto scaling. Differentially accumulated metabolites (DAMs) were screened by combining Student’s *t*-test and OPLS-DA. Metabolites with *p* < 0.05 and log2|FC| ≥ 1 were defined as DAMs. FDR correction was not applied in DAM screening, and the final DAM results were comprehensively interpreted based on FC, VIP values, QC quality and pathway enrichment characteristics. DAMs were further subjected to KEGG pathway enrichment analysis using KEGG database (Release 118.0, http://www.genome.jp/kegg/ (accessed on 6 May 2026)).

### 2.5. ATP Content and ROS Level Assays

ATP content was measured using an enhanced Elabscience^®^ ATP chemiluminescence assay kit (Elabscience, Wuhan, China). Briefly, fresh *C. militaris* mycelium (0.1 g) was homogenized on ice with 0.9 mL of Kit Reagent B, maintaining a tissue weight-to-reagent volume ratio of 1:9 (g:mL). The homogenate was incubated in a boiling water bath for 3 min and rapidly cooled to room temperature with running water. Subsequently, luminescence signals were detected with an MD SpectraMax iD5 microplate reader [26].

Intracellular ROS accumulation was quantified using a Fungal Reactive Oxygen Species (ROS) Assay Kit (BB-461742; BestBio, Shanghai, China). Mycelia of 12-day-cultured CMM and CMKM strains were collected by centrifugation at 5000 rpm for 10 min, washed three times with PBS, and stained with the kit-provided fluorescent solution at 37 °C for 30–45 min in the dark. After staining, mycelial pellets were resuspended in PBS, and the suspensions were loaded into black 96-well plates. Fluorescence intensity was detected at excitation/emission wavelengths of 510 nm and 610 nm using a microplate reader for ROS level quantification [27].

### 2.6. Integrated Transcriptome–Metabolome Analysis

Transcriptome and metabolome datasets from the identical CMKM vs. CMM comparison were integrated at the pathway and correlation levels [28]. Pearson correlation analysis was performed to evaluate the associations between gene expression levels and metabolite abundances. Correlations with |r| > 0.8 and *p* < 0.05 were considered significant. Significant correlation pairs were used to build clustered heatmaps and interaction networks, in which nodes represent DEGs/DAMs and edges represent correlation strength and direction. Clustered heatmaps and correlation networks were ultimately constructed to reveal coordinated gene–metabolite regulatory patterns governing PR development and bioactive metabolite production in *C. militaris* [29].

### 2.7. qRT–PCR Validation of Transcriptome Sequencing Data

Representative DEGs identified in this study were selected for qRT–PCR verification. The gene-specific primers used for analysis were synthesized by Sangon Biotech Co., Ltd. (Shanghai, China) and are listed in Supplementary Table S1. We used 18S rRNA as the internal reference gene [30], and the relative gene expression levels were quantified using the 2^−ΔΔCT^ method. Three independent biological replicates were set for each experimental group.

### 2.8. Statistical Analysis

All statistical analyses were performed using GraphPad Prism 9 (GraphPad Software, San Diego, CA, USA), with *p* < 0.05 defined as the threshold of statistical significance. Bars with different letters indicate significant differences at *p* < 0.05. One-way analysis of variance (ANOVA) followed by Tukey’s multiple comparisons test and Student’s *t*-test were adopted for statistical significance evaluation. Significance thresholds were set as *p* < 0.05 (*), *p* < 0.01 (**), and *p* < 0.001 (***).

## 3. Results

### 3.1. ACAD Deletion Blocks Normal PR and Fruiting-Body Development in C. militaris

**Figure 1:**
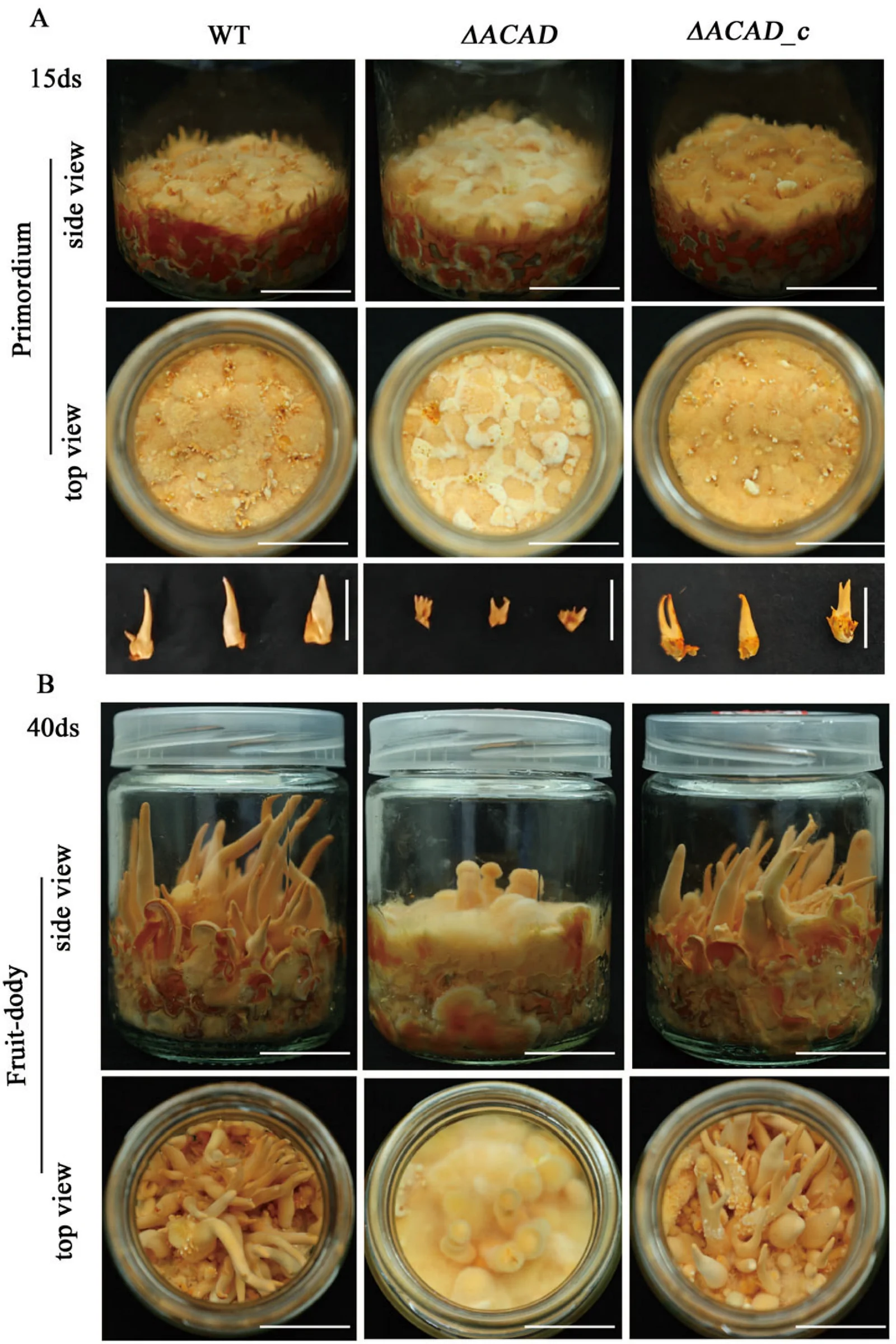
Phenotypic observation of PR and fruiting-body development in WT, Δ*ACAD* mutant and complementary strain Δ*ACAD*_c. (**A**) Morphological characteristics of PR after 15 days of cultivation. The growth status of PR was photographed from side view and top view (scale = 1 cm), and isolated PR were isolated for detailed morphological comparison (scale = 0.5 cm). (**B**) Fruiting-body phenotypes after 40 days of cultivation. The growth status of PR was photographed from side view and top view (scale = 1 cm).

To elucidate the biological role of *ACAD*, we compared the developmental phenotypes of the wild-type (WT) and Δ*ACAD* mutant strains. After 14 days of cultivation on wheat medium under a 12 h light/12 h dark photoperiod, WT mycelia successfully differentiated into PR. The Δ*ACAD* mutants produced malformed and stunted PR (Figure 1A). Following 40 days of cultivation, WT strains produced abundant, well-developed fruiting bodies with elongated stipes and fully mature fertile tissues (Figure 1B). By comparison, the Δ*ACAD* mutants formed only swollen, aberrant PR-like structures with severely stunted stipes. The complementary strain Δ*ACAD*_c restored the normal PR and fruiting-body phenotypes. These phenotypic differences indicated that *ACAD* is an essential positive regulator of normal PR initiation and subsequent fruiting-body development in *C. militaris*. To further elucidate the molecular basis of these developmental defects, we performed integrated transcriptomic and metabolomic analyses to characterize the regulatory networks perturbed by *ACAD* deletion.

### 3.2. Transcriptomic Quality Control and Multivariate Statistical Analysis

**Figure 2:**
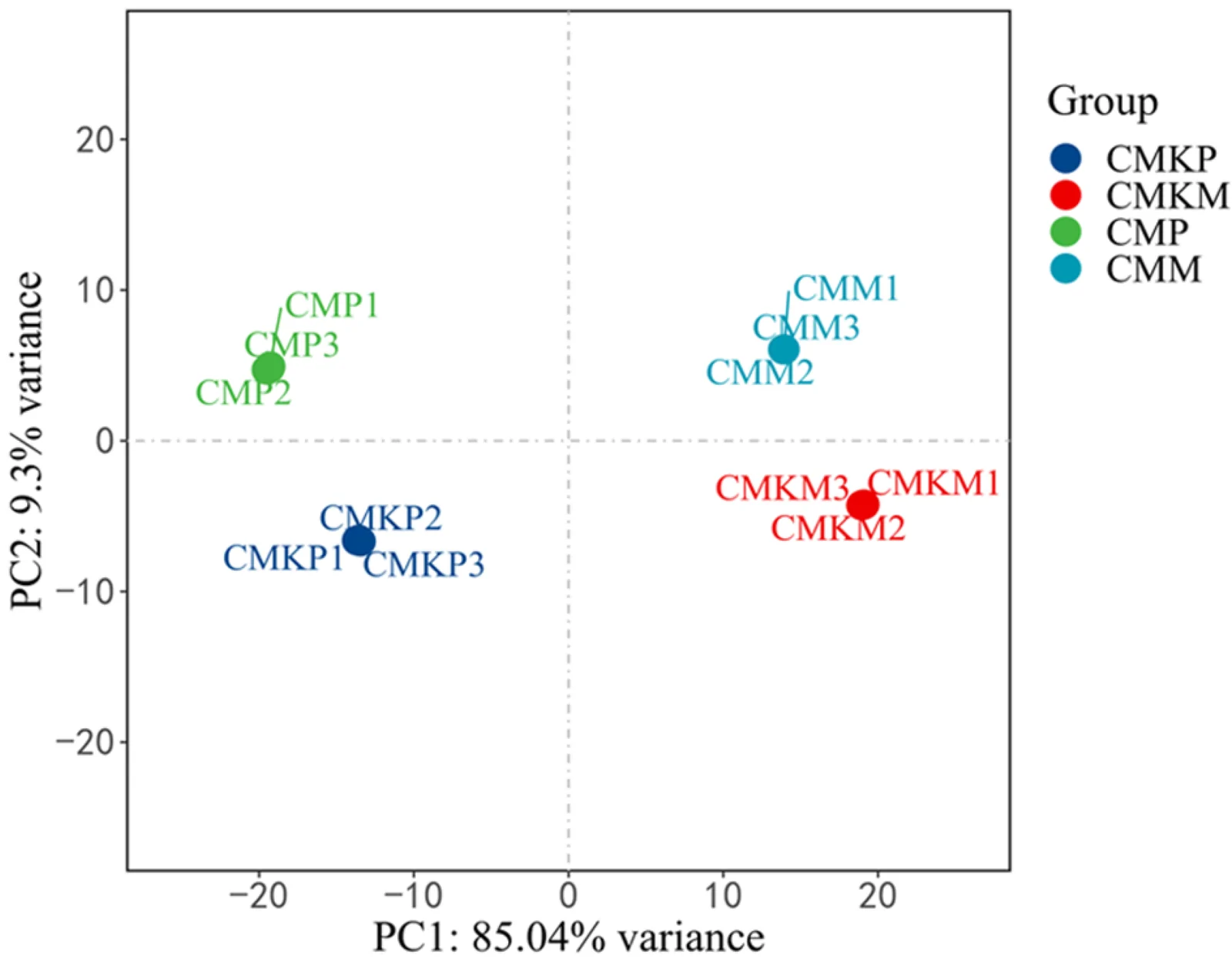
Principal component analysis (PCA) score plot displaying transcriptomic separation among CMKM, CMM, CMP, and CMKP.

A total of 12 transcriptome samples were sequenced in this study. The raw reads of each sample ranged from 23.46 M to 24.10 M; after quality filtering, all samples yielded over 23.16 M clean reads, with valid base proportions between 98.20% and 98.76%. All samples had Q30 values above 97.49%, and GC contents stayed stably within 57.15–57.58% with negligible intergroup fluctuations, collectively verifying robust sequencing data quality. Principal component analysis (PCA) was subsequently performed to assess transcriptional variation across WT and Δ*ACAD* strains at the mycelial and PR stages. The top two principal components, PC1 and PC2, accounted for 85.04% and 9.3% of the total transcriptional variance, respectively (Figure 2). Clear sample separation was observed based on developmental stage and genotype: vegetative mycelia samples (CMM and CMKM) formed one tight cluster, whereas PR samples (CMP and CMKP) assembled into a distinct separate group, revealing that developmental stage-specific transcriptional programs dominate transcriptomic divergence between strains, while *ACAD* knockout induces substantial transcriptional shifts within the same developmental phase, jointly shaping the overall transcriptional divergence across strains.

### 3.3. Differential Gene Expression

**Figure 3:**
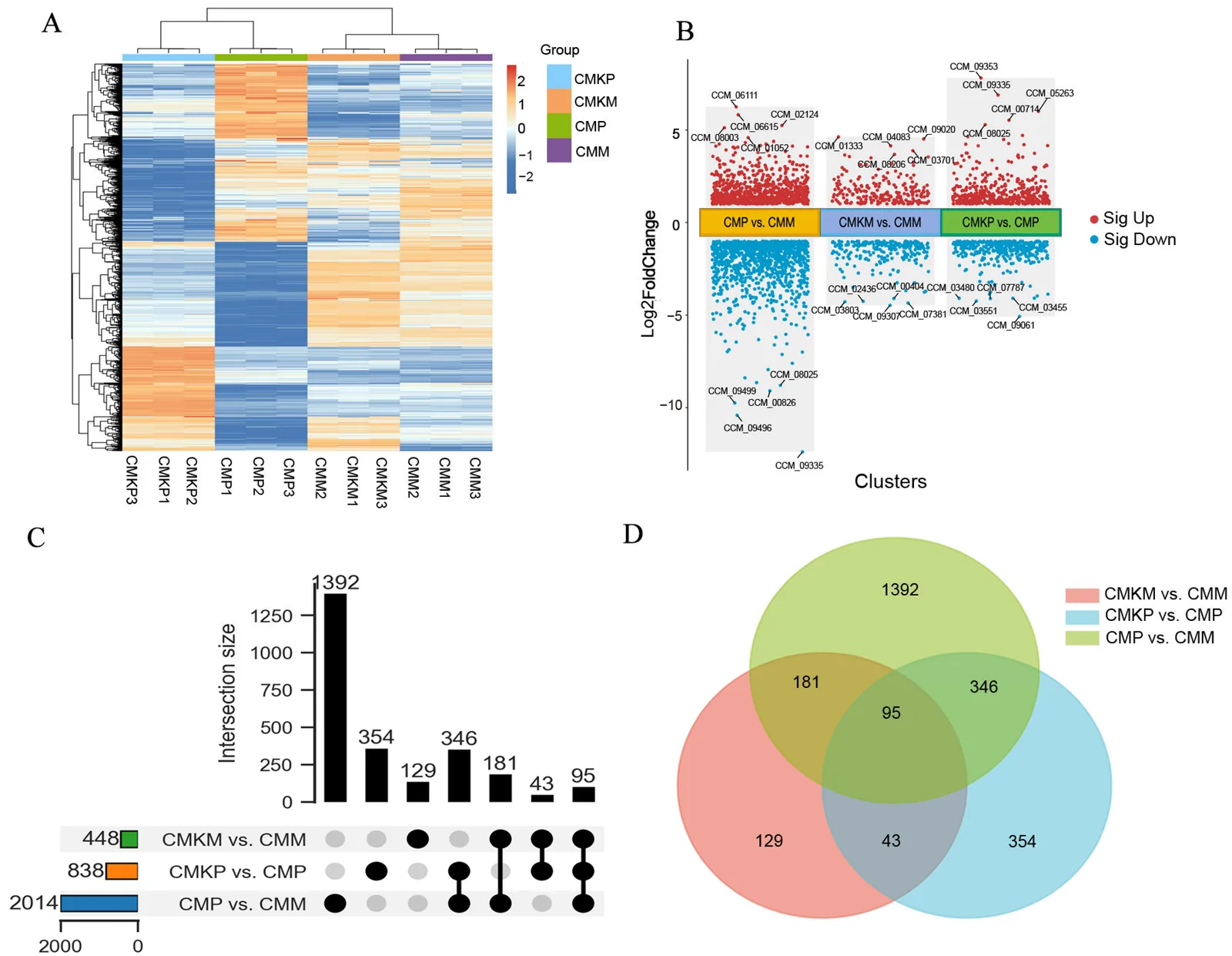
Overview of differentially expressed genes analysis in the comparison groups of CMP vs. CMM, CMKM vs. CMM and CMKP vs. CMP. (**A**) Hierarchical clustering heatmap based on the relative abundances of DEGs. (**B**) Volcano plots displaying DEGs identified from these comparisons. (**C**) UpSet plot showing the shared and specific DEGs among comparison groups. (**D**) Venn diagram showing the shared and unique DEGs between selected comparison groups.

Three pairwise comparisons were performed: CMP vs. CMM, CMKM vs. CMM, and CMKP vs. CMP. We identified 2014, 448 and 838 DEGs in the three groups, respectively. Hierarchical clustering based on normalized expression levels of DEGs showed that all biological replicates within each group clustered tightly together, and samples were distinctly separated by both developmental stage and *ACAD* depletion. CMM and CMKM formed separate clusters, whereas CMP and CMKP constituted another two independent subgroups (Figure 3A). This result indicates that obvious transcriptional divergence occurs both between the mycelial and PR stages and upon *ACAD* deletion.

Volcano plot analysis identified abundant DEGs across all three comparison groups (Figure 3B, Table S2). In CMP vs. CMM, 1167 DEGs were downregulated, and 847 were upregulated. In CMKP vs. CMP, 385 DEGs were downregulated, and 453 were upregulated. In CMKM vs. CMM, 225 were downregulated, and 223 were upregulated.

UpSet analysis and a Venn diagram further visualized the shared and unique DEGs among the comparison groups (Figure 3C,D, Table S3). A total of 1392 unique DEGs were identified in the CMP vs. CMM group, 129 unique DEGs in the CMKM vs. CMM group, and 354 unique DEGs in the CMKP vs. CMP group. Among all overlapping sets across pairwise comparisons, 346 DEGs were shared by the CMP vs. CMM and CMKP vs. CMP comparisons, 181 DEGs were shared by the CMP vs. CMM and CMKM vs. CMM comparisons, and 95 DEGs overlapped exclusively across all three pairwise comparisons.

### 3.4. Functional Enrichment Analysis

**Figure 4:**
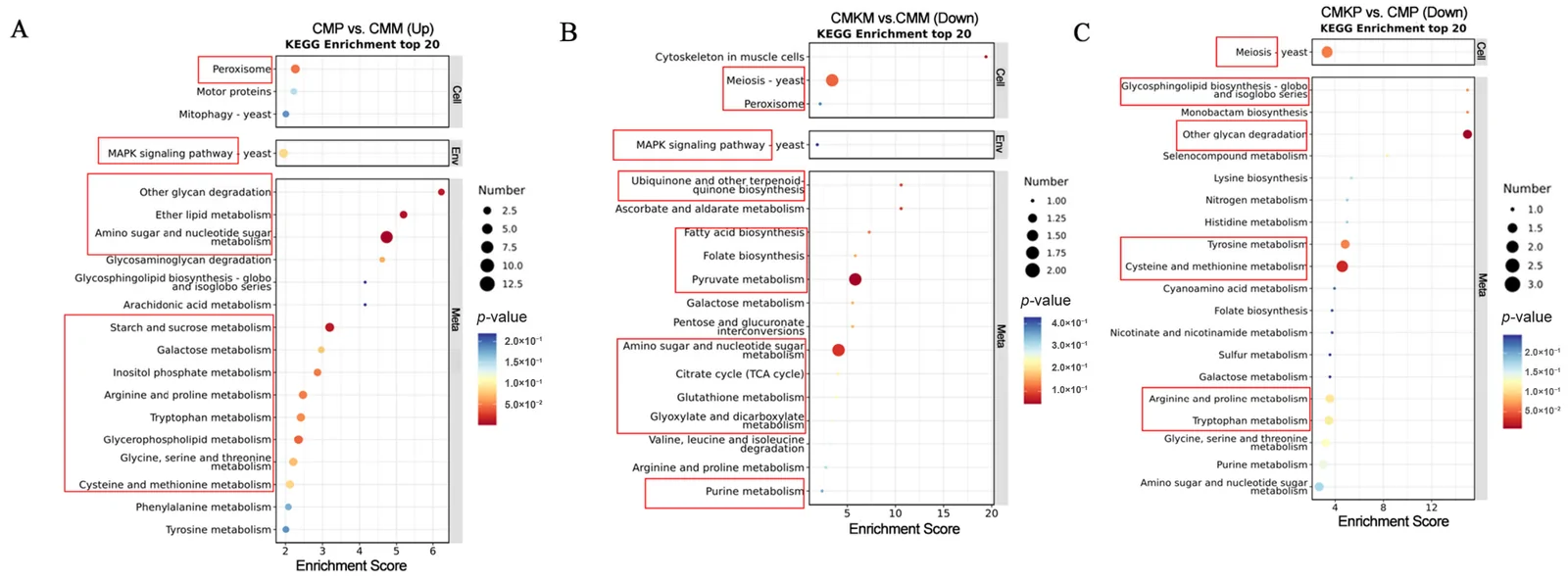
KEGG pathway enrichment analysis of the DEGs from the three comparisons. (**A**) KEGG pathway enrichment bubble plot of the significantly upregulated DEGs in CMP vs. CMM. (**B**) The KEGG pathway bubble plot of the significantly downregulated DEGs in CMKM vs. CMM. (**C**) KEGG pathway enrichment bubble plot of the significantly downregulated DEGs in CMKP vs. CMP. The size of each bubble represents the gene number enriched in the pathway, and the color corresponds to the *p*-value. The red boxes denote key representative KEGG pathways selected for further discussion.

These significantly upregulated DEGs in CMP vs. CMM were tightly associated with PR morphogenesis in *C. militaris*. The KEGG pathway enrichment analysis of upregulated DEGs from the CMP vs. CMM comparison revealed distinct functional enrichment patterns (Figure 4A, Table S4). The highly enriched functional pathways, including cellular process pathways (e.g., peroxisome (cmt04146), motor proteins (cmt04814)), environmental signal transduction pathways (e.g., yeast MAPK signaling pathway (cmt04011)) and metabolic pathways (e.g., ether lipid metabolism (cmt00565); amino sugar and nucleotide sugar metabolism (cmt00520); starch and sucrose metabolism (cmt00500); arginine, proline and tryptophan metabolism (cmt00330); Inositol phosphate metabolism (cmt00562); and glycerophospholipid metabolism (cmt00564)), are shown in Figure 4A, Table S4. The upregulation of these pathways in CMP indicates that coordinated processes, including MAPK signaling, cell cycle progression, cell differentiation, plasma membrane maintenance, amino acid metabolism, nucleotide synthesis, and ROS homeostasis, promote PR morphogenesis in *C. militaris.*

The downregulated DEGs identified in CMKM vs. CMM serve as the downstream regulatory targets of *ACAD*. In this comparison, the significantly downregulated DEGs were enriched in multiple pathways, including cellular process (yeast meiosis (cmt04113), peroxisome (cmt04146)), signal transduction pathways (MAPK signaling pathway (cmt04011)), and metabolic pathways (fatty biosynthesis (cmt00061), folate biosynthesis (cmt00790), pyruvate metabolism (cmt00620), Galactose metabolism (cmt00052), TCA cycle (cmt00020), glutathione metabolism (cmt00480), Glyoxylate metabolism (cmt00630), and purine metabolism (cmt00230)), as shown in Figure 4B, Table S4. The suppression of these pathways in *ACAD*-deficient mutants validates that *ACAD* functions as a positive regulator of genes associated with cell cycle progression, cell differentiation, nucleotide synthesis, redox homeostasis, and energy metabolism during PR formation in *C. militaris*.

In CMKP vs. CMP, the significantly downregulated DEGs were enriched in multiple functional pathways, including cellular process (e.g., yeast meiosis (cmt04113)) and metabolic pathways (e.g., glycosphingolipid biosynthesis (cmt00603), histidine metabolism (cmt00340), tyrosine metabolism (cmt00350), cysteine and methionine metabolism (cmt00270), folate biosynthesis (cmt00790), sulfur metabolism (cmt00920), galactose metabolism (cmt00052), arginine and proline metabolism (cmt00330), and purine metabolism (cmt00230)), as shown in Figure 4C, Table S4. The broad downregulations of these pathways in *ACAD* deletion strains indicate that *ACAD* positively modulates genes governing cell cycle progression, cell differentiation, plasma membrane integrity, amino acid metabolism, and nucleotide synthesis during PR morphogenesis.

**Figure 5:**
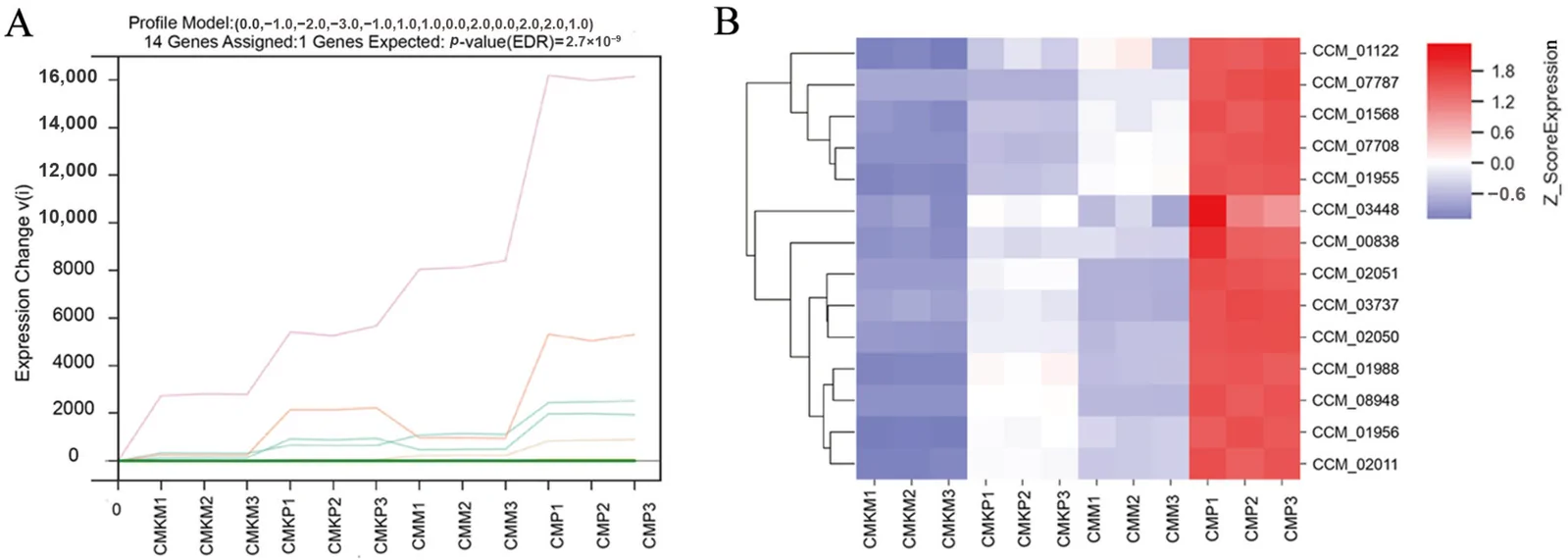
Expression trend analysis and expression heatmap of the 14 DEGs (**A**) Expression-trend profile of the 14 DEGs, each colored line corresponds to the expression trajectory of one individual gene. The x-axis represents sequential sample groups across CMKM, CMKP, CMM and CMP series, the y-axis shows expression change v(i). (**B**) Expression heatmap of the 14 genes selected from the 95 shared DEGs across the CMKM, CMM, CMKP, and CMP sample series.

Moreover, the 95 overlapping DEGs identified from the three pairwise comparisons (CMP vs. CMM, CMKM vs. CMM, and CMKP vs. CMP) were closely linked to PR development that is simultaneously regulated by ACAD in *C. militaris*. Functional enrichment analysis indicated that these overlapping DEGs were predominantly in oxidative phosphorylation (cmt00190), glycolysis/gluconeogenesis (cmt00010), TCA cycle (cmt00020), glyoxylate and dicarboxylate metabolism (cmt00630), pyruvate metabolism (cmt00620), peroxisome function (cmt04146), and folate biosynthesis (cmt00790). Trend analysis of these 95 shared DEGs further screened 14 DEGs with an extremely significant adjusted *p*-value of 2.7 × 10^−9^ (Figure 5A). Trend curves and Z-score normalized heatmaps (Figure 5A,B, Table S5) displayed consistent expression patterns. All 14 genes exhibited an upward expression trend during the transition from vegetative mycelium to PR stage. Specifically, these genes presented the highest expression abundance in CMP. In contrast, their expression levels were markedly lower in CMM and were significantly suppressed in CMKM and CMKP. These expression characteristics indicate that *ACAD* deficiency severely inhibits expression of these genes during PR development. The DEGs included GTP cyclohydrolase I (CCM_02050), histone lysine N-methyltransferase ASHR1 (CCM_01956), and adenine phosphoribosyltransferase 1 (APT1, CCM_02051).

Together, significant transcriptomic differences were detected between mycelia and PR and between the WT and *ACAD*-knockout backgrounds. These data indicate that there is a transition from vegetative mycelia to PR-coordinated reprogramming of multiple metabolic pathways, which is largely regulated by *ACAD*. Moreover, in CMKM vs. CMM, multiple pathways critical for PR development, including meiosis, glycosphingolipid biosynthesis, purine metabolism and amino acid metabolism, were significantly downregulated. These results indicate that *ACAD* deficiency in mycelia would subsequently impair PR development by disrupting these development-related metabolic pathways.

### 3.5. Construction and Analysis of Weighted Protein Co-Expression Network (WGCNA) Analysis and PPI Network Analysis

**Figure 6:**
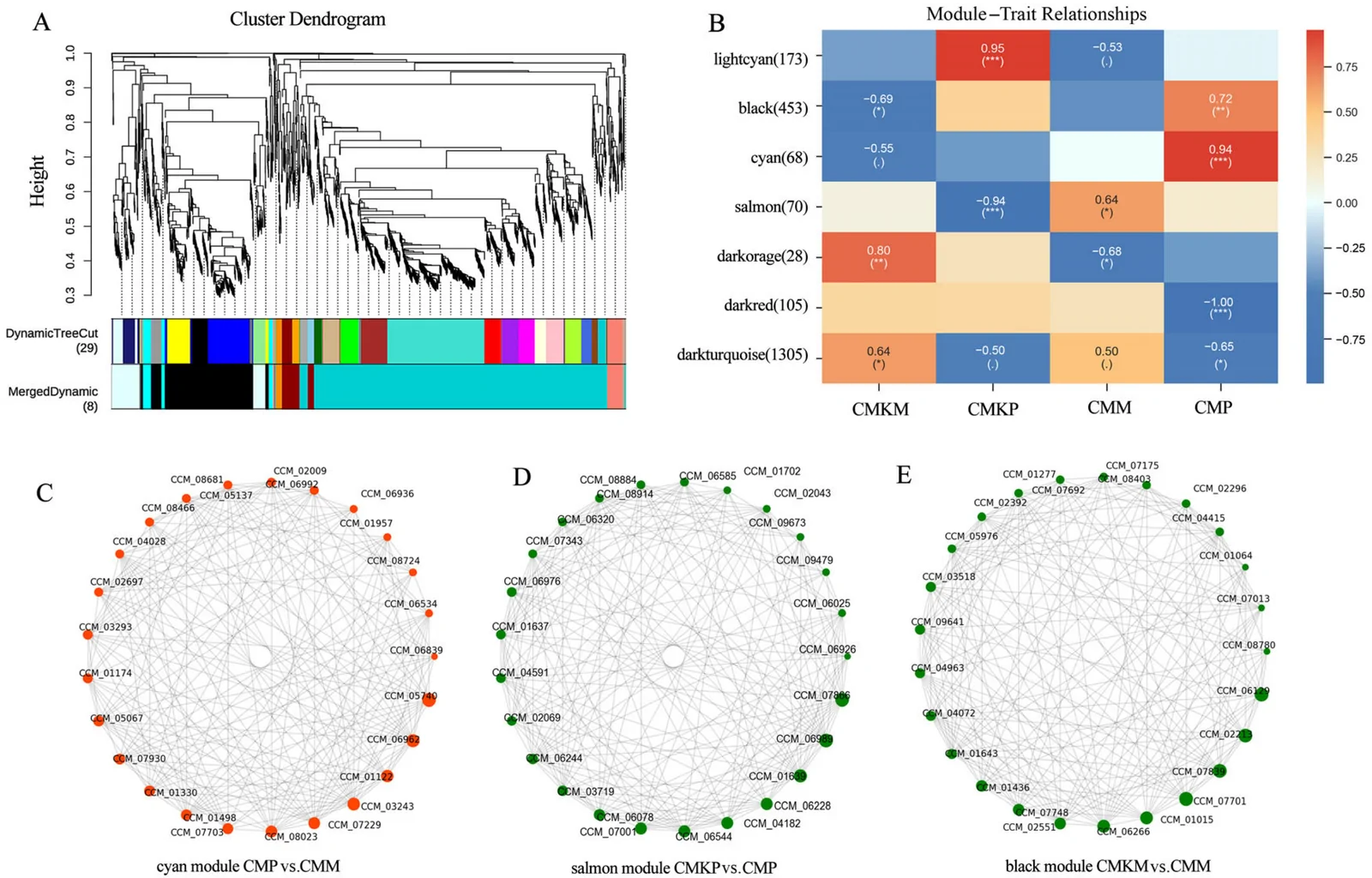
Weighted gene Co-expression network analysis based on transcriptomic data. (**A**) *WGCNA* gene cluster dendrogram showing co-expressed gene modules; Each colored block represents a gene module. The “DynamicTreeCut” row shows the initial module partition, while the “MergedDynamic” row shows the merged modules after merging similar modules. Different colors indicate distinct gene modules. (**B**) Module–trait relationship heatmap illustrating correlation coefficients between each gene module and sample groups (CMKM, CMKP, CMM, CMP). Each row corresponds to a module and each column corresponds to sample group. Blue colors indicate negative correlations, and red values indicate positive correlations: *p* < 0.1, *: *p* < 0.05, **: *p* < 0.01, ***: *p* < 0.001. (**C**–**E**) Gene co-expression network of the cyan module (CMP vs. CMM), salmon module (CMKP vs. CMP) and black module (CMKM vs. CMM). Orange nodes indicate significantly up-regulated genes in the CMP vs. CMM comparison, and green nodes represent significantly down-regulated genes in the CMKP vs. CMP and CMKM vs. CMM comparisons. Nodes represent core genes within the module, and lines indicate gene co-expression relationships.

A WGCNA was constructed to dissect the correlations between gene modules and developmental traits. All expressed genes were clustered into eight distinct co-expression modules (Figure 6A, Table S6). Module–trait–correlation heatmaps revealed that the cyan module was positively correlated with PR formation (*p* < 0.05); the salmon module was negatively correlated with *ACAD* deletion at the PR stage; and the black module was negatively correlated with *ACAD* deletion at the mycelial stage (Figure 6B, Table S6). The cyan module exhibited a positive correlation with PR formation (*p* < 0.05), the salmon module exhibited a negative correlation with *ACAD* deletion in mycelia; and the black module exhibited a negative correlation with *ACAD* deletion during PR formation (Figure 6B, Table S6). Genes within these modules were subsequently extracted for further hub gene screening.

Protein–protein interaction (PPI) networks were constructed to identify hub proteins in the cyan, salmon, and black co-expression modules using an interaction score cutoff of 0.4. The cyan module (CMP vs. CMM) comprising 25 DEGs, the salmon module (CMKP vs. CMP) containing 28 DEGs, and the black module (CMKM vs. CMM) with 25 DEGs all formed densely, highly, and topologically interconnected protein–protein interaction networks, respectively (Figure 6C–E), with corresponding STRING annotations of these proteins listed in Table S7. Dense overlapping edges were observed throughout the network, reflecting high topological connectivity and close functional linkage between all constituent proteins.

Within the cyan module, the major hub genes with high connectivity included probable sphingolipid transporter spinster homolog 1 (Spns, CCM_01330), probable thimet oligopeptidase (CCM_07703), probable CoA-ligase CCL8 (CCM_07930), and probable protein-phosphatase 2C (PP2C, CCM_06936) (Figure 6C). These hub genes were positively associated with PR formation and predominantly involved in central carbon, lipid, sulfur, and purine metabolism.

In the salmon module, principal hub genes included glutathione-S-transferase F3 (GSTF3, CCM_06544), mitochondrial isovaleryl-CoA dehydrogenase (CCM_03805), lysine-specific demethylase JMJ30 (CCM_04182), oligopeptide transporter 5 (CCM_06585), purine-uracil permease NCS1 (CCM_01637), ribosome-inactivating-protein lychnin (CCM_09479), sex-determination-protein tasselseed-2 (TS2, CCM_05246), thiamine-thiazole synthase (TTS, CCM_01639), and ubiquitin-carboxyl-terminal-hydrolase 3 (CCM_06926). These hub genes were consistently downregulated following *ACAD* deletion and formed a moderately connected interaction network (Figure 6D). Moreover, these hub genes are predominantly involved in folate biosynthesis, phenylalanine, tyrosine and tryptophan biosynthesis, N-glycan biosynthesis, and the MAPK signaling pathway. The genes are subcellularly localized to cellular compartments, including the cell wall, endoplasmic reticulum (ER), and chloroplast. These hub genes coordinately regulate energy metabolism, redox homeostasis, cell wall remodeling, and developmental signal transduction.

For the black module, the highest-connectivity hub genes included berberine-bridge-enzyme-like protein (CCM_04963), cytochrome P450 (CYP450, CCM_03518), heat-shock-protein 90 (HSP90, CCM_07839), cyprosin (CCM_01643), glutamate-carboxypeptidase LAMP1 (CCM_05496), serine/threonine-protein-kinase MHK (CCM_01064), DNA photolyase (CCM_02392), and MAPKKK (CCM_02296) (Figure 6E). These hub genes were predominantly involved in starch and sucrose metabolism, phenylalanine/tyrosine/tryptophan biosynthesis, protein processing in N-glycan biosynthesis, galactose metabolism, MAPK signaling pathway and tyrosine metabolism.

### 3.6. Determination of ROS Levels and ATP Contents in CMKM vs. CMM

**Figure 7:**
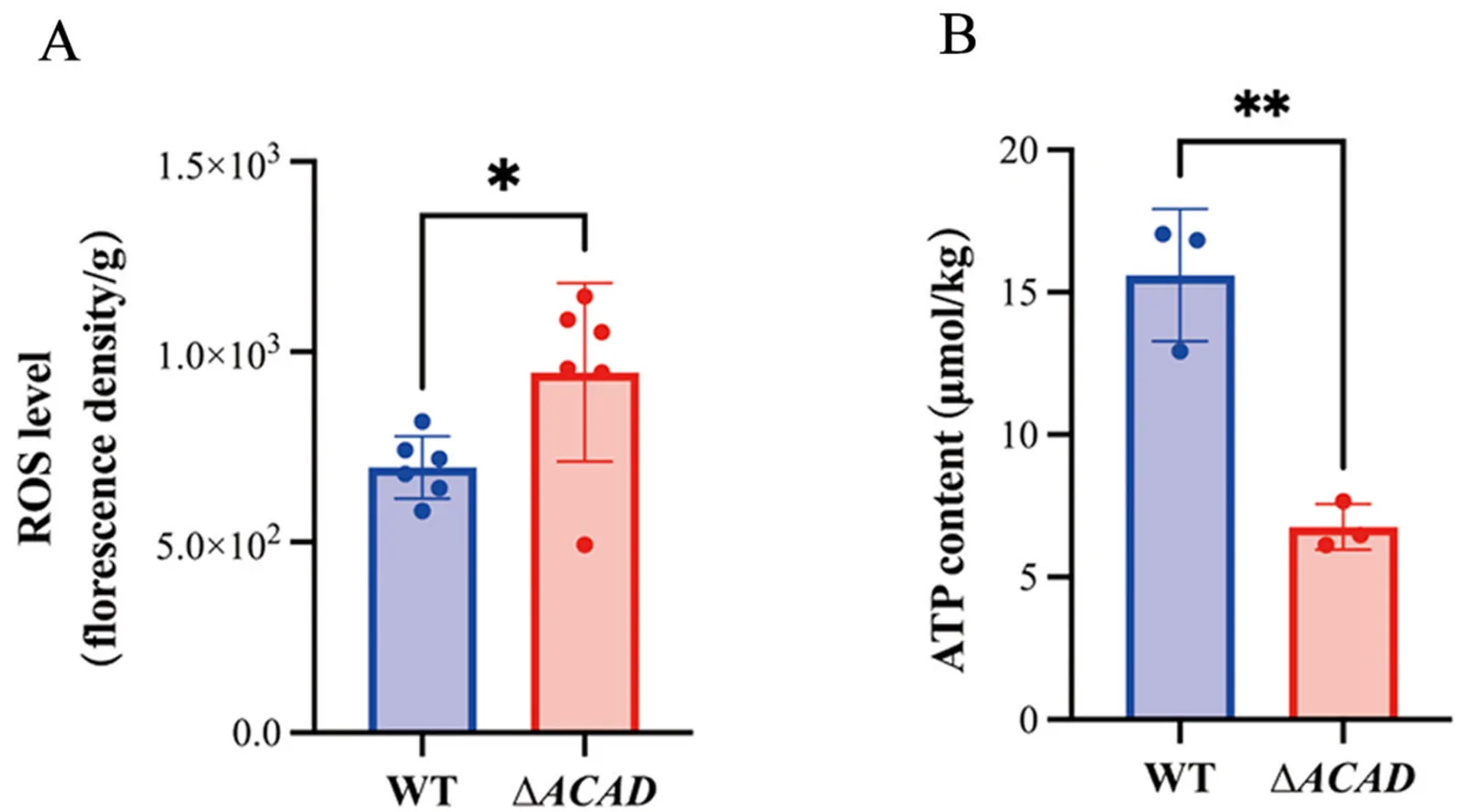
Determination of ROS levels and ATP content in CMKM vs. CMM. (**A**) Measurement of ROS level (fluorescent density/g) in CMKM vs. CMM. *: *p* < 0.05. (**B**) Measurement of ATP content (μmol/kg) in CMKM vs. CMM. **: *p* < 0.01. Data in FG are shown as mean ± SD (*n* ≥ 3), with individual data points overlaid on the bar graphs.

To evaluate intracellular ROS accumulation and energy status between the *ACAD*-knockout and WT mycelia, intracellular ROS levels and ATP contents were quantified. The results showed that the intracellular ROS level in CMKM was approximately 1.35-fold higher than that in CMM (*p* < 0.05) (Figure 7A). By contrast, the ATP content in CMKM was markedly reduced, accounting for only 36% of that in CMM (*p* < 0.01) (Figure 7B). Taken together, the loss of *ACAD* triggered excessive intracellular ROS accumulation, accompanied by a sharp decline in cellular ATP production in mycelia.

### 3.7. Metabolomic Quality Control and Multivariate Statistical Analysis

**Figure 8:**
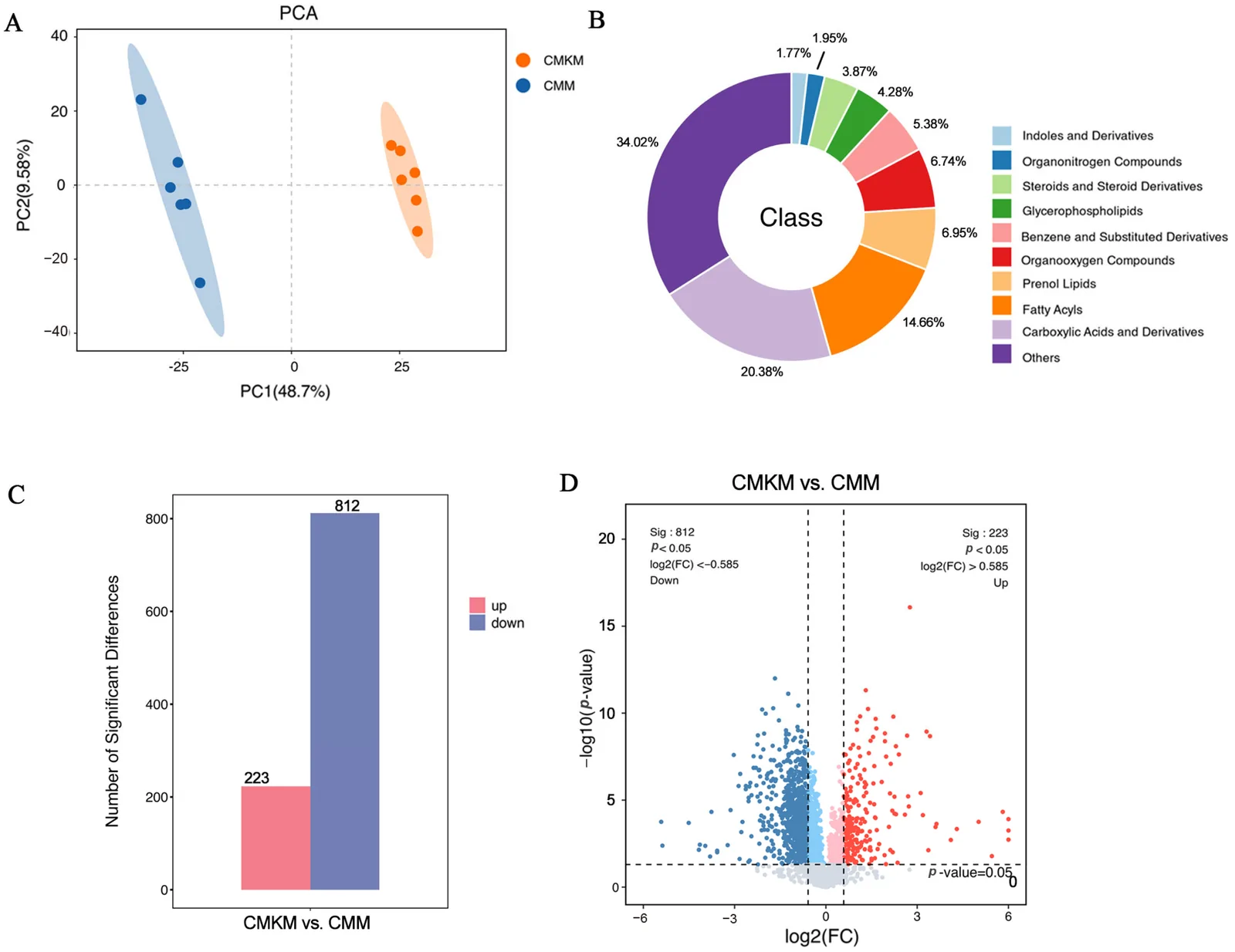
Overview of metabolomic profiling and differential metabolite analysis between CMKM and CMM groups (**A**) PCA score plot displaying metabolic separation between CMKM and CMM groups. (**B**) Pie chart showing chemical classification of all detected metabolites. (**C**) Bar chart summarizing the numbers of significantly upregulated and downregulated metabolites in CMKM vs. CMM. (**D**) Volcano plot illustrating log_2_FC and log_10_(*p*-value) distribution of all metabolites to identify DAMs between CMKM and CMM.

In positive ion mode, the total ion chromatograms (TICs) of the quality control (QC) samples showed high consistency in peak shape, peak intensity, and retention time, indicating good repeatability of the sample extraction procedure and stability of the LC–MS system. PCA showed that the first two principal components, PC1 and PC2, explained 48.7% and 9.58% of the total variance, respectively (Figure 8A, Table S8), indicating distinct separation of CMKM and CMM. The QC samples were tightly clustered in the PCA score plot, further confirming the reliability of the metabolomic dataset and the stability of the instrumental analysis.

Chemical classification of the detected metabolites showed that the predominant classes included carboxylic acids and derivatives (20.38%), fatty acyls (14.66%), prenol lipids (6.95%), organooxygen compounds (6.74%), benzene and substituted derivatives (5.38%), glycerophospholipids (4.28%), and steroids and steroid derivatives (3.87%) (Figure 7B). Minor categories with relatively low proportions consisted of organonitrogen compounds (1.95%) and indoles and derivatives (1.77%). Overall, the metabolite pool of *C. militaris* exhibited broad chemical diversity, where carboxylic acid derivatives, fatty acyl lipids and prenol lipids constituted the core dominant metabolite subclasses. Volcano plot analysis showed that many differential metabolites were detected in each comparison group. In CMKM and CMM, 1035 differential metabolites were identified, including 223 upregulated and 812 downregulated metabolites (Figure 8C,D, Table S9). These data indicate that the *ACAD* deficiency broadly suppresses SM biosynthesis at the mycelial stage, which subsequently impairs PR formation.

### 3.8. Differential Metabolites and Pathway Enrichment Analysis

**Figure 9:**
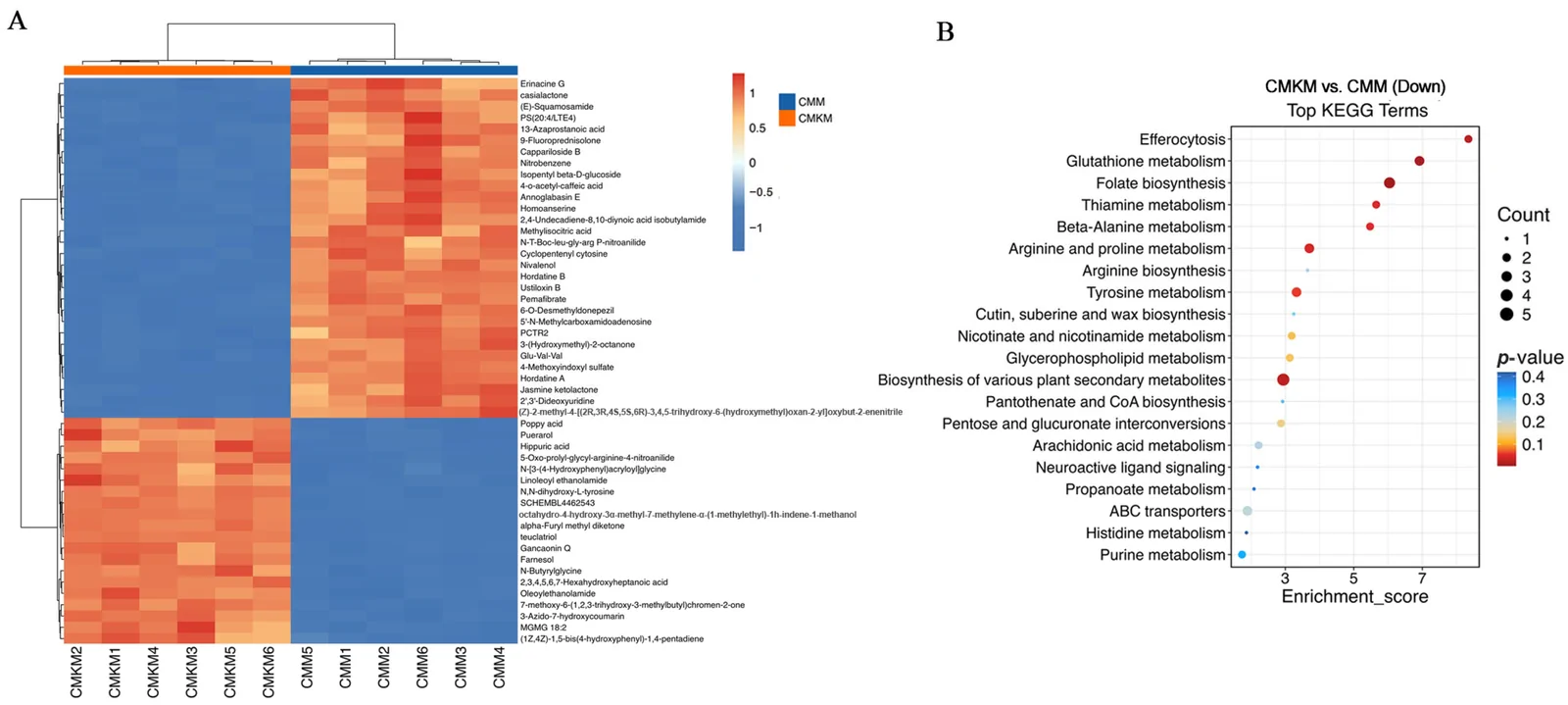
Expression patterns of significant DAMs and KEGG enrichment analysis of these downregulated metabolites in CMKM vs. CMM (**A**) Expression heatmap of all significant DAMs in CMKM vs. CMM. Red indicates higher relative metabolite abundance, while blue represents lower abundance in CMKM compared to CMM. (**B**) Bubble plot showing top 20 significantly enriched KEGG pathways of downregulated metabolites in CMKM relative to CMM. The x-axis is the enrichment score, dot size corresponds to the number of enriched metabolites, and color gradient reflects the *p*-value.

To characterize the metabolic differences between WT and Δ*ACAD* strains in mycelia, the DAMs were screened based on the |log_2_FC| ≥ 1 and statistical significance. A cohort of DAMs that were markedly downregulated in CMKM strains compared to CMM includes: terpenoid and alkaloid SMs (e.g., erinacine G, (E)-squamosamide, nivalenol, hordatine A/B, ustiloxin B), polyunsaturated lipid derivatives (e.g., acylcarnitines (ACs) esters, PS (20:4/LTE4), phosphatidylcholine (PC) (2:0/2:0), and phosphatidic acid (PA) (22:2/0:0), 13-azaprostanoic acid, and amide (E)-squamosamid), phenolic acid and flavonoid glycoside compounds (e.g., 4-o-acetyl-caffeic acid, cappariloside B, isopentyl-D-glucoside), the nucleoside-related metabolites (e.g., 2′,3′-dideoxyuridine (ddl), 5′-amino-5′-deoxyadenosine (5′-AdA)), amino acid derivatives (e.g., homoanserine, Glu-Val-Val and Boc-modified arginine-containing peptides), fatty acyls (e.g., acylcarnitines (ACs) esters, 13-azaprostanoic acid amide (E)-squamosamid, and Oxylipins), and aromatic heterocyclic small molecules (e.g., jasmine ketolactone, 4-methoxyindoxyl sulfate), as well as nucleosides and their analogs (e.g., 5′-AdA, ddl, puromycin aminonucleoside, adenylosuccinic acid (S-AMP) and 2′,3′-dideoxyribonucleosides ddNs) (Figure 9A, Table S10). These results revealed widespread downregulation of nucleoside-, fatty-acyl-, and amino acid-derived metabolites in mycelia following *ACAD* deletion, which might subsequently impair PR initiation and the accumulation of bioactive metabolites.

Further KEGG pathway enrichment analysis of downregulated DAMs in CMKM vs. CMM revealed multiple significantly enriched pathways (*p* < 0.05) (Figure 9B), including efferocytosis, Glu metabolism, folate biosynthesis, thiamine metabolism, pantothenate and CoA biosynthesis, arginine and proline metabolism, and purine metabolism. Hence, *ACAD* deletion triggered this metabolic reprogramming, which may impair carbon flux distribution, energy supply, and ROS homeostasis, consequently restricting SM biosynthesis during fungal development.

### 3.9. Integrated Transcriptome–Metabolome Coordination Network and Its Implications

**Figure 10:**
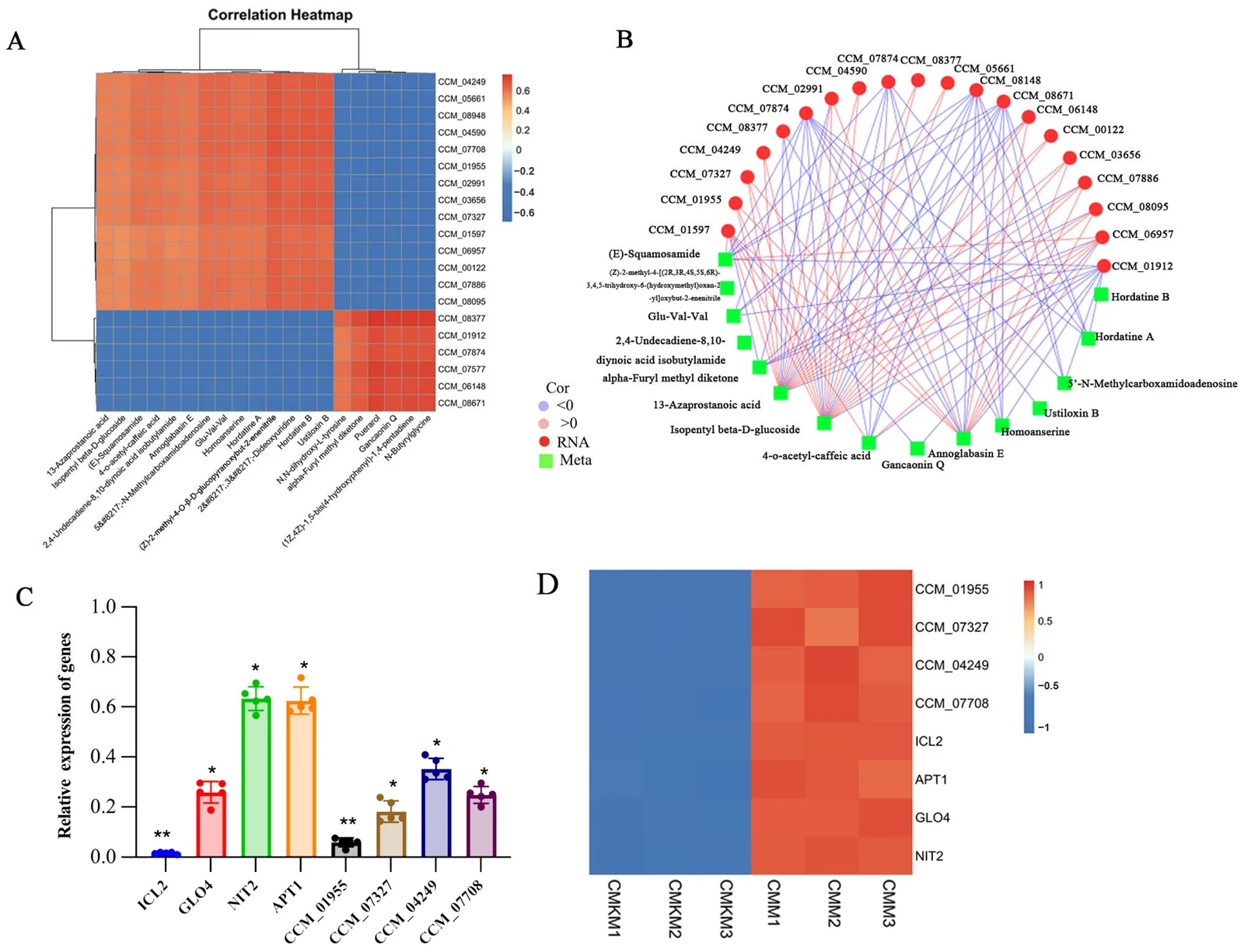
Integrated transcriptome–metabolome correlation analysis in CMKM vs. CMM. (**A**) Correlation heatmap showing pairwise correlation coefficients between key DEGs and DAMs in CMKM vs. CMM. The color gradient from red to blue represents positive to negative correlation, respectively. (**B**) Transcriptome–metabolome correlation network in CMKM vs. CMM. (**C**) qPCR was used to verify the expression levels of the selected DEGs in CMKM vs. CMM. All the data are expressed as the average value of three biological replicates, the line represents the standard deviation, and *p* < 0.05 was considered to indicate statistical significance. (**D**) Heatmap of the selected DEGs from the CMKM vs. CMM comparison based on transcriptomic data *: *p* < 0.05, **: *p* < 0.01. ICL2: Isocitrate Lyase 2, GLO4: Peroxisomal (S)-2-hydroxyacid oxidase GLO4, NIT2: Nitrilase 2, APT1: Adenine Phosphoribosyltransferase.

To systematically characterize the molecular relationship between CMKM and CMM, an integrated gene–metabolite correlation network was constructed using the shared DEGs and DAMs identified between the comparison groups. A correlation analysis was constructed using the shared DEGs and DAMs screened from the comparison of CMKM vs. CMM, shown in the heat map (Figure 10A). The gene–metabolite correlation network exhibited extensive connectivity, highlighting the close coordination between transcriptional reprogramming and metabolic remodeling in mycelia and in response to *ACAD* deletion (Figure 10B).

At the metabolite level, numerous DAMs, including lipid derivatives, glycosides, short peptides, phenolic acids, amino acid metabolites, nucleosides and diverse fungal SMs, occupied central positions within the transcriptome–metabolome network and showed extensive correlations with multiple hub DEGs. Among these, the lipid metabolite 13-azaprostanoic acid, (E)-squamosamid and isopentyl β-D-glucoside displayed the highest connectivity, demonstrating strong associations with nearly all the identified DEGs, suggesting that lipid metabolism, carbohydrate and glycoside metabolism, and nitrogen-containing amide and peptide metabolism are among the metabolic processes most profoundly reprogrammed following *ACAD* deletion during the mycelial stage. Furthermore, several characteristic fungal SMs, including Annoglabasin E, Gancaonin Q, Ustiloxin B, Hordatine A and Hordatine B, showed strong associations with distinct subsets of DEGs. These network relationships demonstrate that *ACAD* deficiency induces extensive remodeling of lipid, carbohydrate, amino acid, phenylpropanoid, purine, and specialized secondary metabolic pathways, all of which are closely linked to PR formation in *C. militaris*.

**Figure 11:**
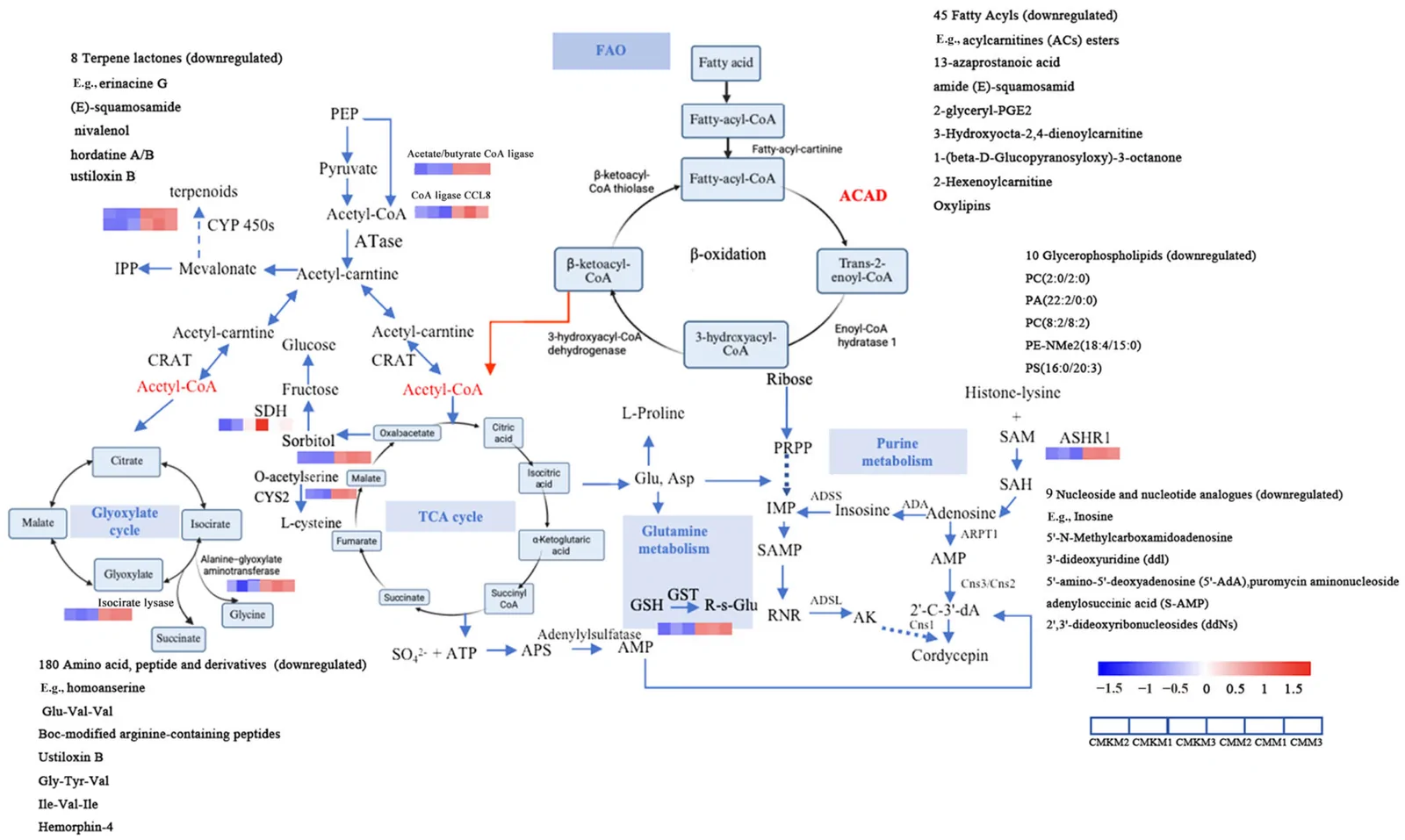
Proposed regulatory metabolic networks underlying ACAD-governed PR development and SM synthesis in *C. militaris*. Black and blue arrows indicate the direction of biochemical reactions and metabolic flux. Red arrows highlight key metabolic-flow rerouting triggered by ACAD deficiency. The small colored bars beside genes represent relative abundance changes in CMKM vs. CMM, corresponding to the color gradient scale at the bottom-right. Blue indicates decreased abundance, red indicates increased abundance in CMKM vs. CMM, ranging from −1.5 to 1.5; bar columns correspond sequentially to CMKM2, CMKM3, CMM2, CMM1, and CMM3. Major classes of down-regulated metabolites are listed on the sides of the diagram. FAO: Fatty acid oxidation, *ACAD*: Acyl-CoA dehydrogenase, CRAT: Carnitine O-acetyltransferase, CYP450s: Cytochrome P450 monooxygenases, IPP: Isopentenyl pyrophosphate, SDH: Succinate dehydrogenase, PEP: Phosphoenolpyruvate, TCA cycle: Tricarboxylic acid cycle, CYS2: Cysteine synthase 2, APS: Adenosine 5′-phosphosulfate, AMP: Adenosine monophosphate, GST: Glutathione S-transferase, GSH: Reduced glutathione, SAM: S-Adenosylmethionine, ASHR1: Histone-lysine N-methyltransferase.

At the transcriptional level, 20 screened core DEGs showed high network connectivity and formed diverse positive and negative correlations with multiple DAMs (Table S11). Three highly connected genes, CCM_01955, CCM_07708, and CCM_08948, remain functionally unannotated. These genes were also included among the 14 DEGs identified through trend analysis, showing significant upregulation during PR development but significant downregulation following *ACAD* deletion. Other metabolite-associated hub genes included peroxisomal (S)-2-hydroxyacid oxidase (GLO4, CCM_02991), isocitrate lyase 2 (ICL2, CCM_05661), nitrilase 2 (NIT2, CCM_07886) and L-ascorbate peroxidase 5 (APX5, CCM_01912). The expression levels of several key DEGs were validated via qPCR (Figure 10C), showing good agreement with transcriptomic profiling results (Figure 10D). The proposed regulatory model illustrating the *ACAD*-modulated PR formation and SM synthesis in *C. militaris* is presented in Figure 11.

## 4. Discussion

By integrating transcriptomic profiling, untargeted metabolomic analysis and phenotypic–biochemical verification, this work explores the complex regulatory networks and crucial genes underlying PR formation and bioactive metabolite biosynthesis in *C. militaris*.

### 4.1. Molecular Differences in PR Development and SM Biosynthesis Between ΔACAD and WT at the Mycelia and PR Stages

*ACAD* deletion severely impaired PR initiation and subsequent fruiting-body formation in *C. militaris*, demonstrating that *ACAD* acts as an essential positive regulator of the vegetative-to-reproductive developmental transition. Integrated transcriptomic and metabolic data showed that the developmental shift from mycelia to PR requires the coordinated activation of a large suite of genes and metabolic pathways rather than alteration of individual genes or metabolites.

To further explore the molecular basis of *ACAD*-regulated PR development and metabolic regulation, three comparative groups (CMP vs. CMM, CMKM vs. CMM, and CMKP vs. CMP) were investigated. Marked activation of these pathways in CMP compared to CMM indicates that coordinated processes, including MAPK signaling, cell cycle progression, cell differentiation, membrane lipid metabolism, amino acid metabolism, nucleotide synthesis, and ROS homeostasis, collectively promote PR morphogenesis. In *Lentinula edodes*, shifts in fatty acyl composition and increased cerebroside accumulation during the PR and fruiting-body stages indicate that lipid metabolism is crucial for fruiting-body morphogenesis [31]. Additionally, amino acid metabolism provides nitrogen sources, carbon skeletons, and metabolic precursors required for fruiting-body development, as well as supporting the synthesis of nitrogen-containing bioactive compounds. Xiang Q. et al. reported significant enrichment of amino acid metabolism during PR formation and fruiting-body development in edible fungi such as *Hypsizygus marmoreus*, highlighting their fundamental role in fungal morphogenesis and SM biosynthesis [32]. Moreover, Fs*ACAD*-2 and Fs*ACAD*-12 are essential for the catabolism of leucine, valine, isoleucine, and tryptophan in *Fusarium sacchari* [33]. The marked repression of these pathways in *∆ACAD* strains indicates that *ACAD* likely promotes both PR development and SM production, largely by modulating amino acid and lipid metabolism in *C. militaris*.

Moreover, the 95 overlapping DEGs identified from the three comparisons were closely linked to PR development and were simultaneously subject to *ACAD* regulation in *C. militaris*, implying that these genes may constitute a core regulatory module linking *ACAD* function to PR development in *C. militaris*. Trend analysis further pinpointed14 genes with highly significant expression changes under developmental transition and *ACAD*-knockout conditions. Among them, GTP cyclohydrolase I catalyzes the first step of folate biosynthesis, and folate derivatives act as essential coenzymes for central carbon metabolism [34]. It promotes the biosynthesis of purine, methionine and thymidylate and mediates a wide range of enzymatic reactions responsible for the transfer, oxidation and reduction of single-carbon units, which is critical for metabolic reprogramming for fungal development [34]. Histone methylation and acetylation epigenetically govern metabolic rewiring, fungal morphogenesis and pathogenicity [35]. APT1 catalyzes the condensation of adenine and phosphoribosyl pyrophosphate (PRPP) to yield AMP [36], thereby boosting ATP production. More recently, it was demonstrated that phase separation of PRPP amidotransferase (PPAT) into dynamic condensates promotes de novo purine synthesis in yeast [37]. These genes were highly upregulated in PR yet sharply suppressed in *ΔACAD* mutants at both the mycelial and PR stages, indicating that these DEGs are crucial for PR development, and their function relies on *ACAD* function.

Furthermore, WGCNA analysis illuminates the regulatory landscape downstream of *ACAD* during the vegetative-to-PR transition. The genes involved in the cyan module were activated during PR formation and function as a core regulatory module driving PR initiation in *C. militaris*. Among them, S1P is a bioactive sphingolipid metabolite that serves as a key signaling molecule governing diverse cellular processes; its transmembrane transport is mediated by the sphingolipid exporter Spinster homolog 2 (Spns2) [38], suggesting that *ACAD* may regulate Spns2 to promote S1P efflux and signal transduction, which further regulates membrane remodeling and lipid homeostasis for PR initiation. Thimet oligopeptidase is a zinc-dependent metalloendopeptidase that degrades short intracellular oligopeptides and recycles free amino acids [39]. The gene was significantly upregulated in PR relative to mycelia, promoting cellular protein turnover and replenishing amino acid pools to support active metabolite biosynthesis during PR initiation. CCL8 converts various carboxylic acids into acyl-CoA derivatives. Its highly increased expression in PR compared to mycelia enlarges the acyl-CoA reservoir and provides abundant precursors to sustain central carbon metabolism and SM synthesis for fruiting-body development. PP2C is an important member of protein phosphatases [40]. Conserved MAPK-binding motifs were detected in PP2C sequences [41], indicating that PP2C is downstream of MAP kinase in the stress-activated signal transduction pathway. Hence, central-carbon, lipid, sulfur, and purine metabolism coordinately provide energy and precursors for PR formation, and some biological processes are controlled by the stress-activated MAPK signaling pathway.

Genes in the salmon module were markedly repressed in *ACAD*-deleted PR compared to WT PR. Among them, TPP functions as an essential coenzyme for multiple key enzymes involved in central carbon metabolism and branched-chain amino acid catabolism [42]. TS2 is a member of the short-chain dehydrogenase/reductase (SDR) superfamily and catalyzes redox reactions targeting carbonyl and steroid substrates [43]. The maize TS2 is essential for stage-specific floral organ abortion [43]. Conserved TS2 homologs modulate developmental-fate determination and hormone signaling across diverse eukaryotes [43]. Significant downregulation of the TS2 gene in CMKP compared to CMP suggests that *ACAD*-mediated TS2 modulates hormone homeostasis and cellular redox balance to coordinate cell differentiation during PR initiation. Downregulation of these hub genes in CMKP strains substantially compromises central metabolic activity, cell wall biogenesis, energy production and intracellular redox balance, ultimately repressing PR development.

The genes involved in the black module were strongly suppressed in CMKM compared to CMM, with the highest connectivity. Among them, DNA photolyase is a light-dependent enzyme that repairs UV-induced DNA lesions by directly reversing pyrimidine dimers [44]. Cryptochrome-like photolyase CryA from *A. nidulans* is light-inducible and acts as a sensor for oxidative stress [44], indicating that DNA damage repair capacity and oxidative stress sensing are impaired under the knockout of ACDC background. CYP450 catalyzes diverse oxidation reactions involved in secondary metabolism, lipid modification and cellular detoxification [45]. Functional studies in *S. scitamineum* have demonstrated that CYP family members participate in sexual development and stress tolerance [8]. For instance, CYP86 modulates the transcription of the developmental regulator SsPRF1 via fatty acid metabolism, while SsCYP64 maintains cell membrane stability, redox homeostasis, and sexual reproduction through regulating linolenyl alcohol accumulation to control fungal mating and filamentation [8]. The CYP450 gene was strongly suppressed in CMKM compared to CMM, indicating that *ACAD*-mediated CYP regulates redox metabolism, lipid remodeling, and stress adaptation for PR initiation.

To verify the effect of *ACAD* deletion on cellular redox status and energy supply, ROS levels and ATP content were assayed in CMKM vs. CMM. It was revealed that the deletion of *ACAD* severely compromised intracellular energy supply and ROS scavenging in mycelia of *C. militaris*. Consistent with the transcriptomic data, the downregulated DEGs in CMKM vs. CMM were enriched in oxidative defense processes, such as Glutathione (Glu) metabolism and peroxisomal function. The identified DEGs include those encoding GST, sorbitol dehydrogenase (SDH) and N-geranyl-L-glutamate oxidase (LGOX), which participate in cellular oxidative defense and ROS homeostasis. For instance, SDH converts glucose to sorbitol and fructose in a reaction, inducing oxidative stress and tissue damage [46]. GSTs conjugate glutathione (GSH) to xenobiotics, participating in ROS homeostasis. GST alpha 1 (GSTA1) expression was negatively related to lipid droplet accumulation in vitro and in vivo, suggesting that GST-regulated redox balance modulates lipid degradation [47]. Hence, *ACAD* deletion disturbs ROS homeostasis through repressing the above redox processes, thereby suppressing PR formation.

Dysregulated redox status and insufficient energy metabolism jointly interfere with multiple biological processes critical for *C. militaris* sexual development [48]. Depleted intracellular ATP restricts energy-dependent biosynthetic reactions, including the synthesis of cordycepin, cell wall chitin and signal lipids required for fruiting-body elongation [49]. This phenotype aligns with previous reports in *Ustilago maydis*, where defective FAO simultaneously elevates cellular ROS and reduces ATP pools, ultimately arresting perithecial and fruiting-body maturation [49]. Consistent with the transcriptomic data, the significantly downregulated DEGs were enriched in the TCA cycle and glyoxylate cycle, including those encoding ICL2, acetate/butyrate–CoA ligase AAE 7, alanine–glyoxylate aminotransferase 2 (AGT) and carboxylase1. So, *ACAD*-mediated FAO acts as an indispensable regulator of the TCA cycle, glyoxylate cycle, and peroxisome and Glu metabolism, thereby maintaining ROS homeostasis and energy generation for PR formation of *C. militaris*.

Based on the above analysis, significant transcriptomic differences were detected during the mycelia-to-PR transition and *ACAD*-knockout genetic backgrounds. *ACAD* functions as an indispensable positive regulator of PR formation in *C. militaris* by coordinating multiple biological processes, such as cell cycle progression, amino acid metabolism, energy metabolism, membrane lipid synthesis, nucleotide metabolism and redox homeostasis. The widespread repression of these pathways in the Δ*ACAD* strains further highlights the central role of *ACAD* in triggering reproductive development and promoting bioactive compound synthesis through the metabolic network.

### 4.2. Molecular Basis of Key Metabolic Pathways and Bioactive Compound Accumulation, and Their Biological Implications

Metabolomic results demonstrated the broad suppression of SM synthesis in Δ*ACAD* mycelia compared to WT. Adenosine and guanosine monophosphates (IMP, AMP and GMP) are important for vegetative growth, sexual/asexual reproduction, and infectious growth, whereas purine salvage synthesis is dispensable for the process in *F. graminearum* [50]. De novo synthesis of GMP is distinct in the sexual stage [50]. Moreover, Acd1, an ortholog of AMP deaminase, is dispensable for growth but essential for ascosporogenesis and pathogenesis, suggesting that purine metabolism has stage-specific functions during sexual reproduction and in-host invasive growth [51]. In the present study, nine nucleosides and their analogs were significantly downregulated in CMKM relative to CMM. Among them, S-AMP, a pivotal intermediate in purine metabolism, stands at the crossroads of energy homeostasis, nucleotide biosynthesis, and cellular signaling. Moreover, these adenosines and their analogs act as the biosynthetic precursors of key bioactive nucleosides in *Cordyceps* spp., such as cordycepin. So, *ACAD* deficiency reduces metabolic substrate supply, inhibiting the production of these bioactive compounds, as well as sexual development in *C. militaris*.

Fatty acid metabolism is essential for cell membrane homeostasis and protection of fungal cells against oxidative stress. Exogenous supplementation with cis-11-eicosenoic acid (C20:1 N9), pentadecanoic acid (C15:0), and linolenic acid (C18:3 N3) significantly restores the mating and filamentous growth of SsCYP86 deletion mutants, demonstrating that SsCyp86-mediated fatty acid metabolism regulates mating competence in *S. scitamineum* [8]. A total of 45 DAMs classified as fatty acyls were markedly downregulated in the mycelia of the Δ*ACAD* mutants relative to WT. Among them were AC esters formed by the conjugation of acyl-CoA with carnitine, which facilitates the translocation of acyl-CoAs across the inner mitochondrial membrane to undergo FAO [51]. These fatty acid metabolites are necessary for cellular energy homeostasis [51]. Oxylipins act as endogenous developmental cues governing hyphal growth, membrane remodeling and redox homeostasis [52]. Additionally, glycosylated lipid conjugates act as storage forms of bioactive lipids and participate in cell interaction [53]. It was implied that *ACAD*-mediated FAO sustains the fatty acyl pool, coordinating membrane integrity, lipid signal transduction and sexual differentiation in *C. militaris*.

Glycerophospholipids serve as fundamental structural and signaling lipids governing hyphal growth and sexual differentiation in fungi. Here, most DAMs classified as glycerophospholipids were markedly downregulated in CMKM compared to CMM. As key membrane components, PA and PC participate in membrane construction and intracellular signal transduction [54]. Hence, *ACAD* deficiency disrupts glycerophospholipid synthesis and compromises membrane homeostasis, thereby impairing the transition from vegetative growth to sexual development.

KEGG pathway enrichment analysis revealed Glu metabolism as one of these core pathways, which tightly governs cellular redox homeostasis and antioxidant defense. The dramatic reduction of lipid peroxidation products resulted in severe oxidative stress and further disturbed Glu metabolism, which resulted from impaired *ACAD* function. Meanwhile, defective folate and thiamine biosynthesis limited the supply of one-carbon units and essential cofactors and suppressed pantothenate and CoA biosynthesis, reducing intracellular acetyl-CoA pools [55]. The suppression of diverse amino acid metabolism and SM biosynthesis collectively disrupts precursors supply for energy turnover, membrane remodeling and developmental differentiation.

Overall, *ACAD* primarily coordinates membrane lipid metabolism, purine metabolism, amino acid metabolism and redox homeostasis, thereby promoting the accumulation of multiple metabolites. These metabolites provide the precursors for the synthesis of key bioactive metabolites, further promoting membrane remodeling and developmental differentiation in *C. militaris*.

### 4.3. Transcriptome–Metabolome Coordination Network

The integrated transcriptome–metabolome correlation network illustrated tight coordination between transcriptional reprogramming and metabolic remodeling after *ACAD* knockout.

At the metabolic level, the DAMs associated with lipid metabolism, carbohydrate and glycoside metabolism, and nitrogen-containing amide and peptide metabolism are profoundly reprogrammed following *ACAD* deletion during the mycelial stage. The disturbance of these metabolic pathways alters the accumulation of key precursor metabolites closely correlated with the hub genes screened in the present study. At the transcriptional level, three highly connected and functionally unannotated genes were also included among the 14 DEGs identified through trend analysis, showing significant upregulation during PR development but significant downregulation following *ACAD* deletion. These uncharacterized genes may function as key regulators that coordinate multiple metabolic pathways required for PR initiation and ensure that the associated metabolic reprogramming is initiated, which requires further study. Moreover, several metabolite-associated hub genes, including GLO4, ICL2, NIT2, and APX5, showed strong positive correlations with multiple DAMs, as shown in Figure 10B. These genes are involved in central carbon metabolism, redox homeostasis, and chromatin modification, along with folate, purine, and energy metabolic pathways. Among them, GLO4 and APX5 were involved in peroxisomal function and functionally associated with redox homeostasis. GLO4 catalyzes the oxidation of various 2-hydroxyacids to corresponding 2-oxoacids coupled with the reduction of O_2_ to H_2_O_2_, exhibiting high catalytic activity toward the long-chain fatty acid 2-hydroxydodecanoate, 2-hydroxyhexanoate and 2-hydroxyoctanoate and (S)-lactate. APX5 belongs to the APX family and is in the peroxisome. APX catalyzes the reduction of H_2_O_2_ to water and plays critical roles in protecting plant cells against stress, as well as in plant growth, development and aging. For instance, TS12 encodes APX5, which is essential for regulating glume development and pollen fertility under heat-stress conditions in rice [56]. In vitro, APXs oxidize non-physiological aromatic substrates, including *p-cresol*, *o-dianisidine*, and guaiacol, at rates comparable to those for ascorbate [57]. Combined with the evidence that FAO occurs exclusively within peroxisomes and participates in fatty-acid catabolism, as well as the biosynthesis of several key phytohormones in plants, including jasmonic acid (JA), indole-3-acetic acid (IAA), and salicylic acid (SA) [58], we speculate that *ACAD*-mediated FAO modulates peroxisome-related metabolic pathways to govern metabolite biosynthesis in *C. militaris*.

ICL2 plays a pivotal role in regulating carbon flux between the TCA cycle, glyoxylate shunt and the methylcitrate cycle at high lipid concentrations, suggesting ICL2 may contribute to energy supply for PR formation in *C. militaris*. NIT2 hydrolyzes nitriles into carboxylic acids and ammonia and plays a key role in hormone biosynthesis and metabolic processes in plants [59]. As a peripheral-membrane protein tightly associated with the plasma membrane, it exhibits the highest expression levels during bolting, flowering, and fruit development [60], suggesting that NIT2 would participate in the regulation of reproductive-related morphogenesis. Here, *NIT2* was significantly downregulated in CMKM relative to CMM and strongly correlated with multiple DAMs, indicating that the loss of *ACAD* repressed *NIT2* expression, which further disturbs nitrile-derived metabolite production and PR development in *C. militaris.* Overall, the integrated network analysis revealed a high degree of transcriptome–metabolome coordination underlying *ACAD*-mediated PR formation and identified candidate regulatory genes and functional metabolites in *C. militaris.*

Overall, under *ACAD*-deficient backgrounds, fungal cells exhibited extensively reprogrammed metabolic networks, which hindered energy supply and redox homeostasis, thereby disrupting SM accumulation and PR formation. These findings provide mechanistic evidence illustrating how *ACAD* governs fungal metabolism and PR morphogenesis and identify key genes potentially targeted by *ACAD* for future functional verification.

## 5. Conclusions

In this study, the regulatory role of *ACAD* in PR formation in *C. militaris* was comprehensively investigated through integrated transcriptomic and metabolomic analyses. The results demonstrated that *ACAD* deletion caused extensive alterations in both gene expression and metabolic profiles. The DEGs and DAMs identified in CMKM vs. CMM were primarily enriched in pathways related to cell cycle regulation, redox homeostasis, and lipid, amino acid and purine metabolism, highlighting the central role of *ACAD* in coordinating developmental progression with metabolic reprogramming. Compared with CMM, CMKM exhibited significantly elevated intracellular ROS and decreased ATP levels. It was revealed that *ACAD* is essential for cellular redox balance and energy supply, which are critical for normal PR development. The gene–metabolite correlation network analysis between *ΔACAD* mutants and WT strains revealed robust transcriptome–metabolome coordination that underpins *ACAD*-modulated PR formation in *C. militaris* and further identified core regulatory genes and functional metabolites governing fruiting-body initiation. Multiple key metabolic pathways, including sucrose metabolism, amino acid metabolism, FAO, glycerophospholipid metabolism and purine metabolism, were markedly suppressed in *ACAD*-deficient mycelia. These findings indicate that extensive transcriptomic and metabolomic alterations triggered by the disruption of *ACAD* occur at the mycelial stage, ultimately impairing subsequent PR formation as well as numerous SM syntheses in *C. militaris*. Future studies focusing on the functional characterization of key genes potentially targeted by *ACAD*, the optimization of cultivation conditions and the synthesis of key bioactive metabolites will further clarify the molecular mechanisms by which *ACAD* regulates PR formation and secondary metabolism, providing a scientific foundation for improving the production of high-quality *C. militaris* and developing value-added functional products.

## Data Availability

The raw transcriptome sequencing data generated in this study have been deposited in the Genome Sequence Archive (GSA) (https://ngdc.cncb.ac.cn/gsa/, accessed on 2 August 2026) under accession number PRJA070389. The metabolomic datasets used in this study are available from the corresponding author upon reasonable request.

## Supplementary Materials

The following supporting information can be downloaded at: https://www.mdpi.com/article/10.3390/jof12090696/s1, Figure S1: PCR amplification of the target gene fragment for Knockout and Complementary Plasmids; Table S1: Primer sequences for plasmid construction and qPCR verification; Table S2: Differentially expressed genes (DEGs) screened from three comparison groups (threshold: q-value < 0.05, |fold change| > 2); Table S3: Upset analysis matrix of DEGs across three comparison groups; Table S4: KEGG pathway enrichment results of the differentially expressed genes in three comparisons; Table S5: Gene expression trend analysis results in CMKM, CMM and CMP; Table S6: Gene module information from WGCNA co-expression network; Table S7: Node and module information of protein–protein interaction (PPI) network from the three modules; Table S8: PCA score matrix of transcriptome samples; Table S9: Volcano plot data of DAMs between CMKM and CMM groups; Table S10: Heatmap data of the top 50 DAMs between CMKM and CMM; Table S11: Top 20 correlation coefficients between key genes and metabolites.

## Abbreviations

WT	Wild-type strain of *Cordyceps militaris*
*ACAD*	Acyl-CoA dehydrogenase
*∆* *ACAD*	*ACAD* gene knockout strain
DEGs	Differentially expressed genes
DAMs	Differentially accumulated metabolites
KEGG	Kyoto Encyclopedia of Genes and Genomes
PPI	Protein–protein interaction
LC–MS	Liquid chromatography–mass spectrometry
QC	Quality control
PCA	Principal component analysis
HEA	N^6^-(2-hydroxyethyl) adenosine
AMP	Adenosine monophosphate
cGMP	Guanosine 3′,5′-cyclic monophosphate
RNR	Ribonucleotide reductase
NT5E	5′-Nucleotidase
ADEK	Adenosine kinase
ROS	Reactive oxygen species
CRAT	Carnitine O-acetyltransferase
CYP450s	Cytochrome P450 monooxygenases
IPP	Isopentenyl pyrophosphate
SDH	Succinate dehydrogenase
CCL8	Acetate/butyrate-CoA ligase
PEP	Phosphoenolpyruvate
TCA cycle	Tricarboxylic acid cycle
CYS2	Cysteine synthase 2
APS	Adenosine 5′-phosphosulfate
GST	Glutathione S-transferase
GSH	Reduced glutathione
SAM	Adenosylmethionine
ASHR1	Histone-lysine N-methyltransferase
PC	phosphatidylcholine
Ddl	2′,3′-dideoxyuridine,
5′-AdA	5′-amino-5′-deoxyadenosine
