# Supplementary material for: Integrated Transcriptomic and Metabolomic Analyses Reveal Acyl-CoA Dehydrogenase-Mediated Primordium Formation and Metabolite Synthesis in Cordyceps militaris
Source: J Fungi (Basel). 2026 Sep 17;12(9):696. doi: 10.3390/jof12090696 (PMC13608744; doi:10.3390/jof12090696)
Supplement: Supplementary file 1 [file jof-12-00696-s001.zip › Tab.S1.pdf]

Table S1. Primer sequences for plasmid construction and qPCR verification

Primer name	Sequences 5'to 3'	Length(kb)	Notes
pEX4-Line-F	CTAGAGTCGACCTGCAGGC	6.7	Linearisation of pEX4-hpt-eGFP
pEX4-Line-R	GAATTCAATTCGGCGTTAATT CAG		
pCT741-Line-F	GGCTATGGGTATGAAGCTGG ATATCTACGTAATGGTGAGC	5.6	Linearisation of pCT741-Neo-eGFP
pCT741-Line-R	ACATGGCAGATGCCGAGCAT AAGCTTGGCCTATATTCATT		
ACAD-up-F	AATTAACGCCGAATTGAATT CGGCCGTACGAGGACGCGC	1.0	Amplification of ACAD upstream
ACAD-up-R	CTACGATCTCGTCGAGGGTC AACGGTGGGTGCACAT		
ACAD-hpt-F	ATGTGCACCCACCGTTGACC CTCGACGAGATCGTAG	1.8	hpt fragment amplification to replace ACAD and identify resistance fragments
ACAD-hpt-R	TGGCGTACTTGACCGACTCC TTAATTCGGGGGATCTGGAT		
ACAD-down-F	ATCCAGATCCCCCGAATTAA GGAGTCGGTCAAGTACGCCA	1.0	Amplification of ACAD downstream
ACAD-down-R	TGCCTGCAGGTCGACTCTAG GGATGACATCGGGTATTAGT		
ACAD-CDS-F	AATGAATATAGGCCAAGCTTA TGCTCGGCATCTGCCATGT	1.8	ACAD backfilling CDS sequence amplification
ACAD-CDS-R	GCTCACCATTACGTAGATATC CAGCTTCATACCCATAGCC		
Neo-F	TATTCTACCCAAGCATCCAAA TGATTGAACAAGATGGATT	0.8	Amplification of Neo-resistant fragments for ACAD backfill
Neo-R	GTTCCCGGTCGGCATCTACTT CAGAAGAACTCGTCAAGAA		
ACAD-F	ATGGCGCTGTCCCGCAGCCT	1.8	Identification of knockout fragment ACAD
ACAD-R	ACCACACGCCCTGGCAGTCC		
CCM-01955-F	TCATGGGAATCGACCTTGGC	0.1	Amplifying the specific fragment of CCM-01955 in *C. militaris*
CCM-01955-R	CTGGCCCTTGGCTAGGAAAA		
CCM-07327-F	TTCCAAGATCACTCCCACCG	0.1	Amplifying the specific fragment of CCM-07327 in *C. militaris*
CCM-07327-R	TTCAGAAGCAGGGCAAGTG G		
CCM-04249-F	TTCAAAGAGGGCGAACCCAT	0.1	Amplifying the specific fragment of CCM-04249 in
CCM-04249-R	CCCGTTTAGGAAGGTGACGA		

			C. militaris
CCM-07708-F	ACCAGCAACGCAACAATCAC	0.1	Amplifying the specific fragment of CCM-07708 in Cordyceps militaris
CCM-07708-R	CCGTACCAGGAGGGGTATGA		
ICL2-F	CCTCTACATTTCCGGCTGGG	0.1	Amplifying the specific fragment of ICL2 in *C. militaris*
ICL2-R	GTGTTGTAGGGGTAGTCGCC		
NIT2-F	GTGATGCTCAGGTCCCCATT	0.1	Amplifying the specific fragment of NIT2 in *C. militaris*
NIT2-R	AGGCTACCGTTGTCGTTACC		
APT1-F	CGATGCTGCTCAAGGCATTC	0.1	Amplifying the specific fragment of APT1 in *C. militaris*
APT1-R	CTGCTCAGGTCCCAGAAAGG		
GLO4-F	CAAGTTTGACGAGCAGGGG T	0.1	Amplifying the specific fragment of GLO4 in *C. militaris*
GLO4-R	GAGGGGTCGATGAAGGAGG A		

The protocol for the generation of the *C. militaris ΔACAD* mutant and its complementary strains

To generate the *C. militaris ΔACAD* mutant, the 1kb upstream and 1kb downstream flanking sequences of *ACAD* (ID: CCM_03805) were amplified from *C. militaris* genomic DNA (ID: NW_006271971) using specific primer pairs *ACAD* -up-F/R and *ACAD* -down-F/R, respectively (Tab. S2). The 1.8 kb hygromycin B phosphotransferase (hpt) resistance gene cassette was amplified from the pEX4-hpt plasmid using primer pairs *ACAD* -hpt-F1/R1. The 6.7 kb pEX4 vector was linearized using primers pEX4-Line-F/R. All amplified fragments were purified using the EasyPure® PCR Purification Kit (TransGene, Beijing, China) and quantified using a NanoDrop One spectrophotometer. The linearized vector, the hpt cassette, and the upstream and downstream flanking fragments of *ACAD* were then assembled into the recombinant plasmid pEX4-hpt-*ACAD* using the in-Fusion® Snap Assembly Master Mix (Takara Biotech, Japan). The recombinant plasmid was verified by sequencing and subsequently transformed into *Agrobacterium tumefaciens* AGL-1 for Agrobacterium-mediated transformation (ATMT) [22]. Transformants were confirmed by PCR. Agarose gel electrophoresis validation of knockout and complementary plasmid construction shown in Fig. S1. All primer pairs used in this study are listed in Tab. S1.

For complementation, the Neo resistance fragment was amplified from pCambia1300-Neo using primers Neo-F and Neo-R, and the PCT741-hpt resistance fragment was replaced with PCT741-Neo. The full-length CDS of ACADs was amplified using ACAD-CDS-F and ACAD-CDS-R, and the PCT741-Neo plasmid was digested with *Hind*III and *EcoR.* The ACAD-CDS was then inserted into the PCT741-Neo vector using the in-Fusion® Snap Assembly Master Mix (Takara Biotech, Japan) to construct the complement plasmid PCT741-Neo-ACAD, PCT741-Neo-ACAD was then introduced into the *ΔACAD* strains by the PEG-mediated protoplast

transformation [23]. Transformants were selected on PPDA agar plates supplemented with 2000 μg/mL of G418and 800μg/mL of hygromycin B at 22 °C. The complemented strain was confirmed by PCR amplification.
